# Self-powered high-sensitivity all-in-one vertical tribo-transistor device for multi-sensing-memory-computing

**DOI:** 10.1038/s41467-022-35628-0

**Published:** 2022-12-23

**Authors:** Yaqian Liu, Di Liu, Changsong Gao, Xianghong Zhang, Rengjian Yu, Xiumei Wang, Enlong Li, Yuanyuan Hu, Tailiang Guo, Huipeng Chen

**Affiliations:** 1 Institute of Optoelectronic Display, National & Local United Engineering Lab of Flat Panel Display Technology, Fuzhou University, Fuzhou, 350002 China; 2 School of Physics and Electronic Engineering, Zhengzhou University of Light Industry, Henan, 450002 China; 3 Fujian Science & Technology Innovation Laboratory for Optoelectronic Information of China, Fuzhou, 350100 China; 4 State Key Laboratory for Chemo/Biosensing and Chemometrics, School of Physics and Electronics, Hunan University, Changsha, 410082 China

**Keywords:** Electronic and spintronic devices, Electronic properties and materials, Electrical and electronic engineering

## Abstract

Devices with sensing-memory-computing capability for the detection, recognition and memorization of real time sensory information could simplify data conversion, transmission, storage, and operations between different blocks in conventional chips, which are invaluable and sought-after to offer critical benefits of accomplishing diverse functions, simple design, and efficient computing simultaneously in the internet of things (IOT) era. Here, we develop a self-powered vertical tribo-transistor (VTT) based on MXenes for multi-sensing-memory-computing function and multi-task emotion recognition, which integrates triboelectric nanogenerator (TENG) and transistor in a single device with the simple configuration of vertical organic field effect transistor (VOFET). The tribo-potential is found to be able to tune ionic migration in insulating layer and Schottky barrier height at the MXene/semiconductor interface, and thus modulate the conductive channel between MXene and drain electrode. Meanwhile, the sensing sensitivity can be significantly improved by 711 times over the single TENG device, and the VTT exhibits excellent multi-sensing-memory-computing function. Importantly, based on this function, the multi-sensing integration and multi-model emotion recognition are constructed, which improves the emotion recognition accuracy up to 94.05% with reliability. This simple structure and self-powered VTT device exhibits high sensitivity, high efficiency and high accuracy, which provides application prospects in future human-mechanical interaction, IOT and high-level intelligence.

## Abstract (web summary)

Designing efficient sensing-memory-computing systems remains a challenge. Here, the authors propose a self-powered vertical tribo-transistor based on MXenes to implement the multi-sensing-memory-computing function and the interaction of multisensory integration.

## Introduction

With the upsurge of artificial intelligence applications and the rapid development of internet of things (IOT), the time-efficient sensing information acquisition, energy-efficient data memorizing and processing capabilities of terminal electronic systems are indispensable^1^. However, in conventional chips, the separated sensing, storage, and processing units always need to collect signals by external sensors, convert signals into digital format data subsequently, and then transfer the date to memory units and processors for subsequent processing tasks, which limits the data conversion and movement and results in low computing efficiency and huge power consumption^2^. Thus, benefiting from in-memory-computing device^3^, which can both store data and compute simultaneously by device physics or other physical laws, an integrated sensing-memory-computing (SMC) system with high-efficiency perception, storage and calculation functions could greatly simplify transmission operations, decrease hardware bulk, and increase efficiency in comparison to the traditional chips^1,4–8^.

Meanwhile, most creatures possess multiple sensory organs with which they can simultaneously perceive a wide variety of physical changes in the environment^9–16^. Thus, SMC systems that can integrate and organize various sensory information (multi-sensing-memory-computing (MSMC)) are fundamental to effective perception and cognitive function^1,4,11,17–20^. Noticeably, MSMC system desires various numbers of sensory receptors and processing nodes (tactile, auditory, and visual, etc) to keep producing multi-sensing raw data and processing different types of sensory information, respectively^15,21–28^. However, the increased number of receptors slows down the working speed due to the subsequent data transmission and processing, and the separated sensory receptors and processing nodes also lead to transmission speed discrepancy, which would further limit the conversion speed and increase energy consumption^24–26^. Therefore, developing a single device integrated with the function of MSMC is of great significance to address the above-mentioned challenges and improve sensory perceptual efficiency in current electronic devices.

Noticeably, triboelectric nanogenerator (TENG) is energy harvesting device that can convert various forms of energy such as human motion, sound vibration, and light energy into electric power^29–36^. The native advantages of TENG render it a promising power supply device as well as a multisensory receptor^33,37–42^. Meanwhile, vertical organic field effect transistors (VOFETs) composed of vertically stacked gate/source/drain electrodes and promising short channel possess small subthreshold swing (SS), high working frequency, and promising mechanical stability, ensuring their great practical applications in memory and computing devices^43–46^. Thus, the inherent vertically stacked electrodes of VOFETs are suitable to integrate with TENG, which possibly provides an effective approach for achieving multi-functional all-in-one-device with sensing-memory-computing function.

Hence, in this work, we develop a vertical tribo-transistor (VTT) device based on TENG and VOFET to implement the multi-sensing-memory-computing function and the interaction of multisensory integration. This VTT is based on a simple configuration of VOFET without any redundant layers, and MXenes function as the top electrode of TENG, source electrode of transistor, and the light collection layer of multisensory, simultaneously. The VTT allows electrostatic induction and tribo-potential to tune ionic migration and Schottky barrier, and thus the triboelectrification sensory information can be amplified to the source-drain current in a self-energy transducing manner, which improves the sensitivity by 711 times over a single TENG. Meanwhile, processing functions of different sensory perception and multisensory interaction in brain superior colliculus are achieved based on the individual VTT device. Furthermore, artificial conscious response is generated by the integration of robot hand with VTT device, and the open angle of robot hand is successfully controlled with different sensory stimulation, demonstrating that this system can straightforward enhance the accuracy of relevant event. Finally, a multi-model emotion recognition system is constructed to detect and distinguish typical mood. The self-powered VTT device shows great potential in next-generation high-performance in-sensor-memory-computing artificial intelligent system and human-computer interaction interface applications.

## Results

### Self-powered vertical tribo-transistor with multi-sensing function

**Fig. 1:**
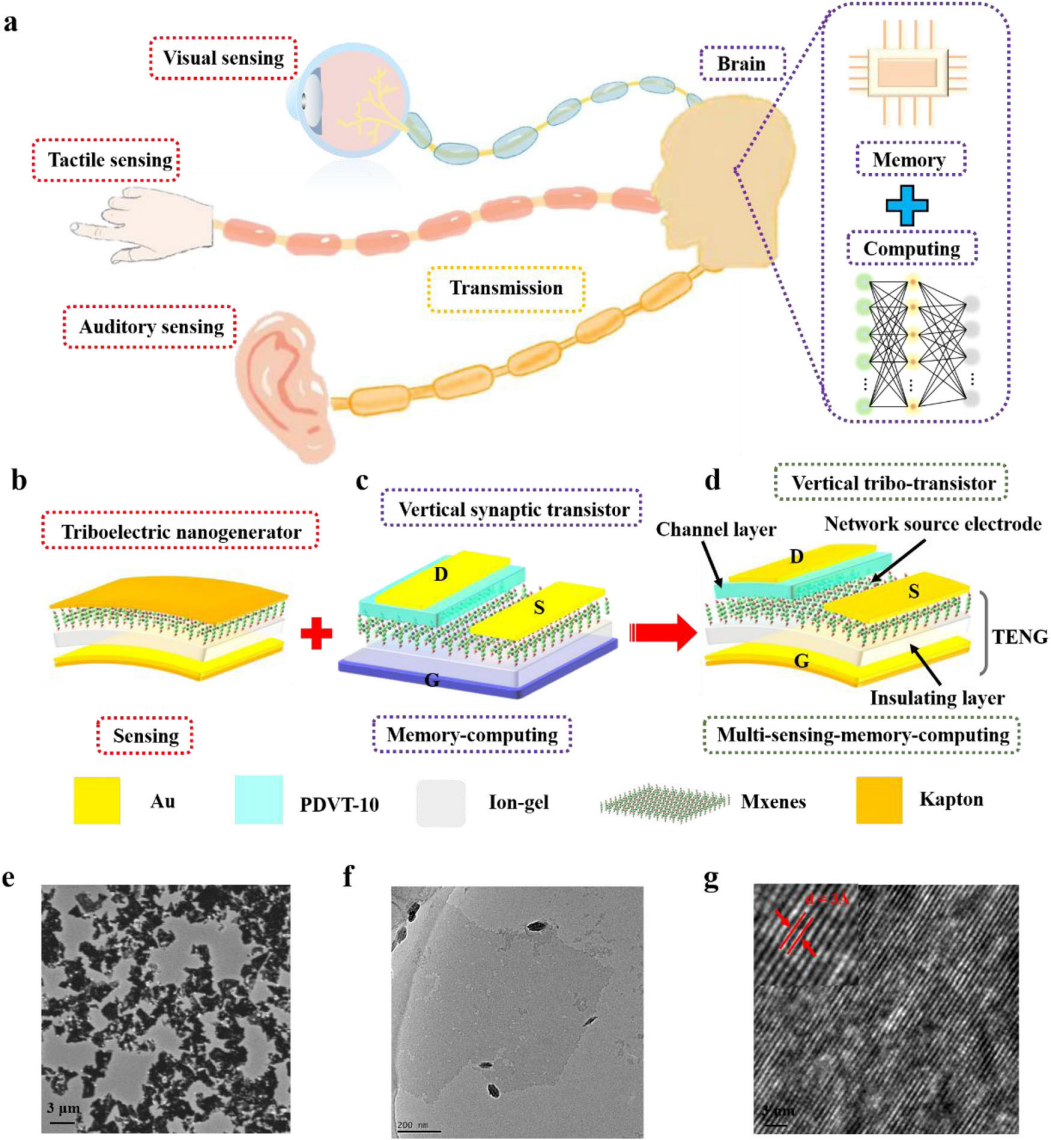
Schematic diagram of multi-sensing-memory-computing system, the device structure of VTT and its morphology characteristics. **a** The biological multi-sensing-memory-computing system. The sensory receptors collect sensory data and transmitted the data to human brain for further memory and computing. **b–d** Schematic illustration the structure of TENG, vertical transistor, and VTT, respectively. **e** SEM images of MXenes network source electrode. **f** SEM images of MXenes network source electrode. **g** High-resolution TEM image of MXenes network source electrode.

A schematic diagram of biological multisensory integration nervous system is illustrated in Fig. 1a. The system is composed of several sensing units for perception, transmission pathways for transfer information, and brain for memory and computing. Here, a vertical tribo-transistor (VTT) device based on TENG (Fig. 1b) and VOFET (Fig. 1c) is fabricated to implement the multi-sensing-memory-computing function, as illustrated in Fig. 1d. Clearly, this VTT integrates the function of a TENG and a VOFET. It is based on a VOFET configuration without any redundant layer, which is attributed to the fact that both TENG device and VOFET device share the vertical structure. The VTT composes removable Kapton substrate, removable Au gate electrode, ion-gel insulating layer, MXenes network source electrode, PDVT-10 channel layer, and Au source/drain electrode. Meanwhile, the detailed fabrication process of VTT is shown in Supplementary Fig. 1, and the cross-section SEM is depicted in Supplementary Figure 2. Noticeably, MXenes, which possess regulated work function, outstanding mechanical and optical properties, have raises significant interest for optoelectronic, energy storage and Schottky-barrier based devices. Thus, MXenes are promising alternative electrode of TENG and network source electrode of VTT here. The sensing abilities, touch and hearing perception are emulated by moving of gate electrode and vibration of VTT, respectively, and the visual perception is achieved by the light-sensitive Ti_3_C_2_T_x_ MXenes electrode.

The scanning electron microscope (SEM) image of MXenes network is presented in Fig. 1e, which clearly shows that the MXenes are interconnected with each other and form a network structure. Meanwhile, the transmission electron microscope (TEM) of MXenes is shown in Fig. 1f, where Mxenes exhibit nanosheets structure, and the thickness of MXenes network source electrode is about 1 nm. Fig 1g shows the high-resolution TEM image, and the corresponding lattice spacing in this layer is 0.3 nm^45^. The electrostatic induction and triboelectrification between gate electrode and insulating layer would induce the electrode-double-layer effects, which manifests in a transient channel current, thus realizing the multi-sensing-memory-computing function.

**Fig. 2:**
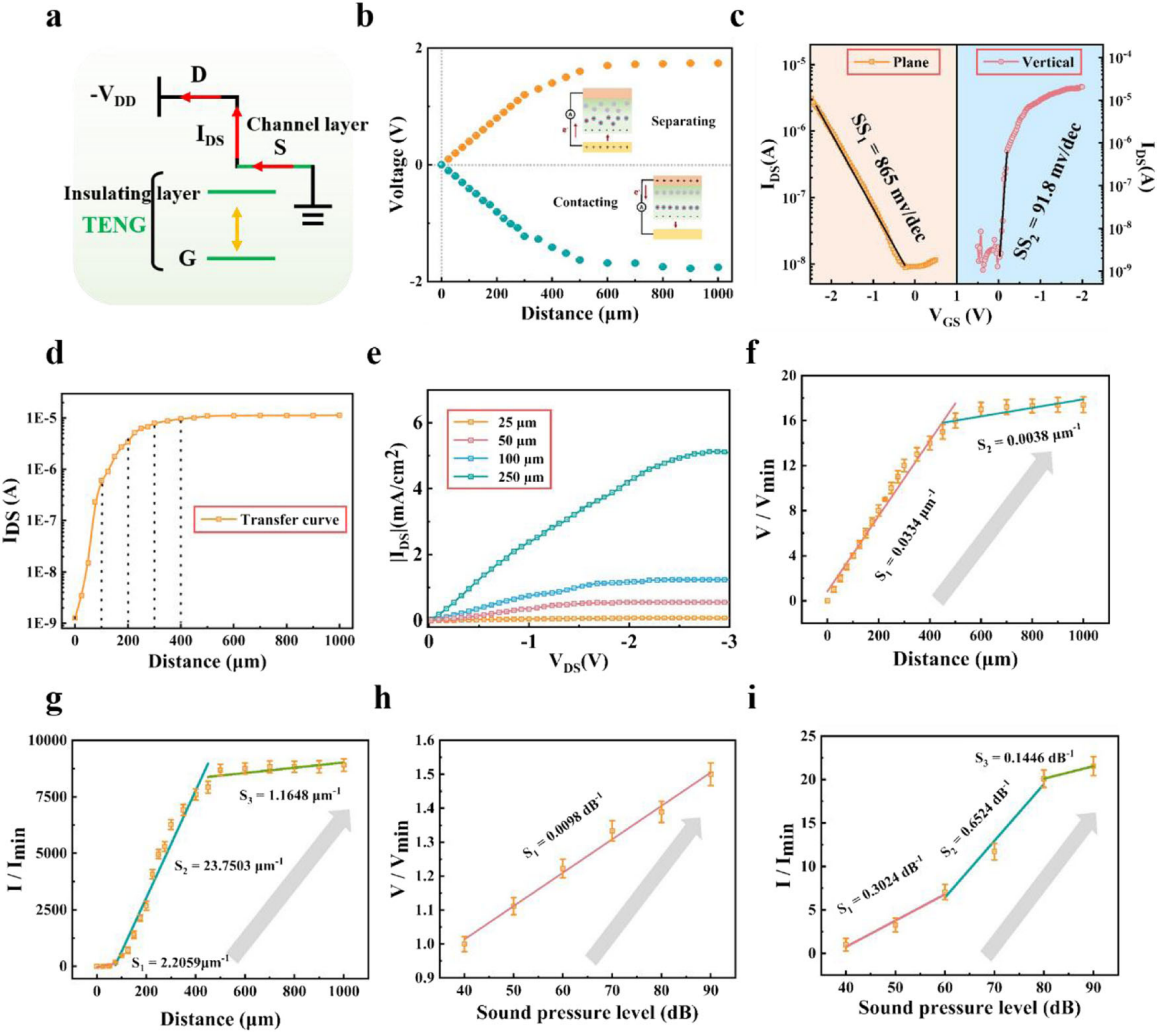
The electrical output and sensing properties of TENG and VTT. **a** Equivalent circuit of the VTT device. **b** The TENG output voltage versus distance. **c** The SS of VTT with external gate voltage. **d**, **e** Transfer curve and output curve of VTT with TENG. **f**, **g** The tactile sensitivity of single TENG and VTT with different distance. **h**, **i** The auditory sensitivity of single TENG and VTT with different sound pressure level. The error bars in f-i means the values of V/Vmin or I/Imin within 10 cycles.

We first investigated the sensing function of this VTT device, and the equivalent circuit of VTT is demonstrated in Fig. 2a. The gate electrode in Fig. 2a can be moved, and the distance between gate electrode and insulating layer is defined as d. Compared with traditional plane transistor integrated with TENG (Supplementary Fig. 3a, 3b), this VTT not only enhances the device integration level, but also simplifies the equivalent circuit (Supplementary Fig. 3). Clearly, VTT can achieve the function of TENG by only leading out the gate and source electrode, while if external voltage is applied to the source-drain electrodes, it can function as VOFET. The output performance of individual TENG is shown in Fig. 2b. With the increase of the distance between gate electrode and insulating layer from 0 to 1000 μm, the open-circuit voltage (V_OC_) increases from 0 to 1.71 V. The working mechanism of TENG is illustrated in Supplementary Fig. 4. Here, ion-gel is employed as the triboelectric material in TENG. The Au electrode and MXenes electrode are employed as the bottom electrode and top electrode in TENG, respectively. When the distance between bottom electrode and triboelectric layer changes, and the confined charges in ion-gel attract the counterions. In the original state, the bottom electrode and ion-gel layer are not in contact with each other, and then when bottom electrode is fully contacted with ion-gel layer (IL), electrons flow to the IL because of the triboelectric effect. When bottom electrode is moved away, positive and negative ions in the IL accumulate at the air/ion-gel and ion-gel/top electrode interfaces, respectively, forming an electrical double layer (EDL). Accordingly, an output voltage is recorded with the electrons flowing from top electrode to bottom electrode. It is noteworthy that the high capacitance of EDL can remarkably improve the performance of TENG (as illustrated in Supplementary Note 1)^37,45,46^.

Then, we investigated the property of the devices as a VOFET. The typical transfer curve of VOFET is illustrated in Fig. 2c, where gate voltage is modulated through external voltage, and the gate electrode is contacted with the insulating layer (Supplementary Fig. 5). From the transfer characteristics of VOFET^43^, the subthreshold swing (SS) of 91.8 mV/dec is extracted, while lower SS would result in higher ΔI (I_DS1_-I_DS2_) at same ΔV (V_GS1_-V_GS2_), which shows the great potential of the device in achieving high sensitivity sensory perception. Noticeably, the V_OC_ of TENG can be considered as a gate-source voltage and then convert into a transient channel current. Figure 2d and Fig. 2e show the transfer and output curve of VTT under different V_OC_ from TENG, respectively. When the distance between gate electrode and insulating layer increases from 0 to 1000 μm (Fig. 2d), the VTT exhibits p-type transistor behavior, and the drain-source current (I_DS_) increases by nearly 4 orders. Moreover, the on-state current density is greater than 5 mA/cm^2^ (Fig. 2e), providing sufficient current density to drive organic electronic devices. This high output current density of VTT is mainly ascribed to its ultrashort channel length. For comparison, the transfer curve of planar transistor is depicted in Fig. 2c left, which shows a higher SS compared with vertical transistor.

Furthermore, we investigated the sensitivity of these two devices individually when they are used for mimicking different sensory perception. Fig 2f, g show the tactile sensitivity of an individual TENG and VTT, respectively. The tactile sensitivity of TENG is defined as equ. 1:

1 $${{{{{\rm{S}}}}}}=(\Delta {{{{{\rm{V}}}}}}/{{{{{{\rm{V}}}}}}}_{{{\min }}})/\Delta {{{{{\rm{d}}}}}}$$

where ΔV is the change of open-circuit V_oc_ (V-V_0_), V_min_ is the minimum V_oc_, and Δd is the changed distance (d) between gate electrode and insulating layer. When d is increased from 0 μm to 450 μm, the sensitivity is calculated to be 0.0334 μm^−^^1^, and then a lower sensitivity of 0.0038 μm^−^^1^ is estimated when the d increases from 450 μm to 1000 μm. In comparison, the tactile sensitivity of VTT is defined as eq. 2:

2 $${{{{{\rm{S}}}}}}=(\Delta {{{{{\rm{I}}}}}}/{{{{{{\rm{I}}}}}}}_{{{\min }}})/\Delta {{{{{\rm{d}}}}}}$$

where ΔI is the change of drain-source current ΔI_DS_ (I-I_0_), I_min_ is the minimum I_sc_, and Δd is the changed distance (d) between gate electrode and insulating layer. As depicted in Fig. 2g, the VTT initially exhibits a sensitivity of 2.2059 μm^−^^1^, and then, when the d is increased from 75 μm to 400 μm, the sensitivity (S_2_) is calculated to be 23.7503 μm^−^^1^, which is 711 times higher than that of the individual TENG. Finally, with the d exceeding 400 μm, which corresponds with a saturated I_DS_ and a lower ΔI, a rapid decrease of the sensitivity to 1.1648 μm^−^^1^ is observed. Thus, different distance between gate electrode and insulating layer would result in different charge distribution, V_oc_, I_DS_, and sensitivity.

Besides, we characterized the auditory and visual stimuli transduction sensitivity of individual TENG and VTT, respectively. For the auditory stimuli transduction sensitivity, a loudspeaker with tunable sound pressure level (SPL) and frequency is used as the acoustic source to trigger the VTT. The auditory sensitivity is defined as eq. 3:

3 $${{{{{\rm{S}}}}}}=(\Delta {{{{{\rm{V}}}}}}(\Delta {{{{{\rm{I}}}}}})/{{{{{{\rm{V}}}}}}}_{{{\min }}}({{{{{{\rm{I}}}}}}}_{{{\min }}}))/\Delta {{{{{\rm{SPL}}}}}}$$

where ΔSPL is the changed sound pressure level of the loudspeaker. As the sound pressure level increases from 40 to 90 dB, the individual TENG device exhibits a nearly linear response and a SPL sensitivity of 0.0098 dB^−^^1^ (Fig. 2h). Meanwhile, the dependence of sensitivity of VTT on SPL is shown in Fig. 2i, and the highest sensitivity is 0.6524 dB^−^^1^ in the range from 60 to 80 dB. Furthermore, the absorbance of MXenes is presented in Supplementary Fig. 6, which exhibits particularly high absorption intensity in ultraviolet (UV) region, and the wavelength light source used is 325 nm next. The photosensitive MXenes function as the top electrode of TENG, source electrode of transistor, and the light collection layer of multisensory, simultaneously, and the visual sensitivity of individual TENG and VTT are illustrated in Supplementary Fig. 7. Clearly, VTT exhibits higher sensitivity than the individual TENG for each sensory perception, and the higher sensitivity of VTT is attributed to the amplification, low SS and high on/off ratio of VOFET. Moreover, the function of each material/layer in VTT with different sensing is shown in Supplementary Fig. 8, which further indicates that this VTT is beneficial to achieve multi-sensing function.

### The working mechanism of self-powered vertical tribo-transistor (VTT)

**Fig. 3:**
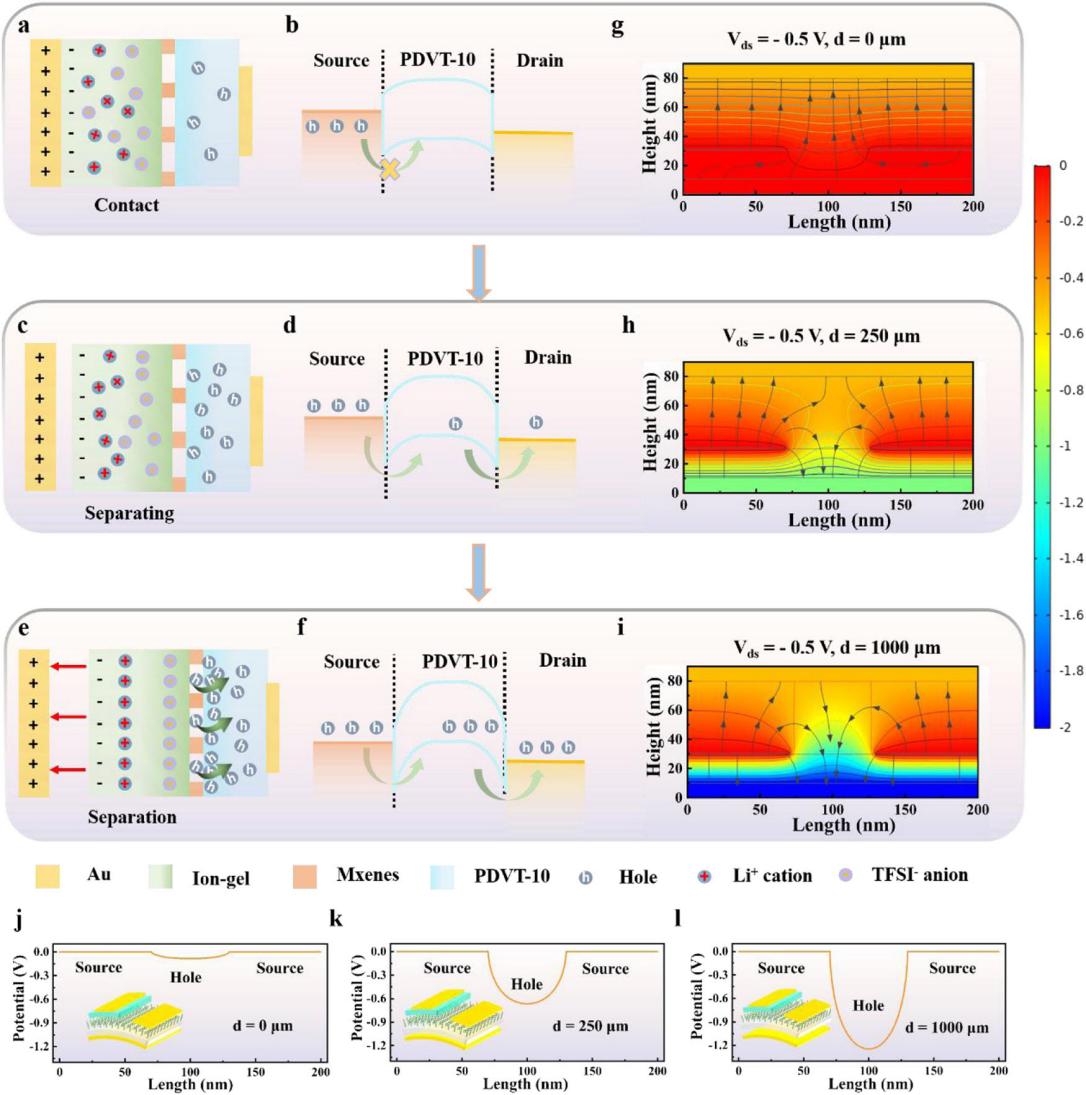
The working mechanism of multi-sensing VTT unit. **a** Charge distribution of the source/channel/drain layers when gate is in contact with the IL layer. **b** Corresponding band diagram of each layer at contact state. **c** Charge distribution of the source/channel/drain layers when gate is separated from the IL layer. **d** Corresponding band diagram of the source/channel/drain layers when gate is separated from the IL layer. **e** Charge distribution of each layer at separation state. **f** Corresponding band diagram of each layer at separation state. **g–i** The electrical field distributions of VTT under three different states. **j–l** Potential distribution at MXenes electrode.

Based on the above results, the working mechanism of VTT unit for tactile signal is illustrated in Fig. 3. In the initial state, the gate electrode is separated with the IL, and then the VTT is set to the original state (the process was depicted Supplementary Fig. 9). As the gate electrode is fully contacted with IL, positive charges and negative charges accumulated on the surface of gate electrode and IL interface because of the electrostatic induction and triboelectrification, respectively, as depict in Fig. 3a. Noticeably, the identical positive charges and negative charges lead to the off state of VTT, and there is no current recorded when a source-drain voltage is applied. The corresponding band diagram of this state is illustrates in Fig. 3b. It is clear that energy band bending would occur at MXenes/PDVT-10 interface when MXenes come into contact with PDVT-10 semiconductor. The high Schottky barrier height between the MXenes and PDVT-10 causes small source-drain current. Next, when the gate electrode and IL are separated, the tribo-potential would regulate the ionic migration of ion-gel layer, and positive ions in the IL are induced based on the charge balance effect (Fig. 3c). Simultaneously, an EDL is gradually generated at the IL, where the number of negative ions balanced the positive charges. Accordingly, temporary holes are accumulated near IL/PDVT-10 interface, which would decrease Schottky barrier height between the IL and PDVT-10, and then a conductive channel from MXene to drain electrode is formed (Fig. 3d). Finally, when gate electrode and IL are further separated, the EDL is entirely generated at the IL because of the tribo-potential, as shown in Fig. 3e. At this stage, massive holes accumulate at the IL/PDVT-10 interface, and thus the width of the Schottky barrier between the HOMO energy level of PDVT-10 and MXenes work function is narrowed, as shown in Fig. 3f, which enables the holes to easily flow into the semiconductor layer. Thus, holes are injected into PDVT-10 semiconductor from MXenes-network source electrode under the source/drain voltage and then are vertically transferred from PDVT-10 channel layer to drain electrode, resulting in the formation of drain-source current (on state).

To further understand the working mechanism and charge transport in VTT, a 2D cross-section simulation model is exploited to demonstrate the electrical field distributions and electrical potential during the aforementioned three states, as depicted in Fig. 3g–l. Notably, the drain-source current of VTT is dependent on the distance between gate electrode and IL, and different distances would result in different gate voltage. Thus, the structure of VTT in COMSOL Multiphysics is simply considered as a typical VOFET with varied gate voltage, as depicted in Supplementary Fig. 10. Figure 3g–i show the electrical field distributions of VTT under three different states, which indicates that the distance between gate electrode and IL could effectively control the charge behavior at the IL/PDVT-10 interface. Furthermore, Fig. 3j–l show the 1D potential distribution of MXenes electrode position (Supplementary Fig. 10). It is observed that the potential becomes more negative as the distance increased, indicating stronger band-bending at the Mxene/semiconductor interface, which is favorable for the injection of holes into the PDVT-10 channel. Moreover, the working mechanism of acoustic signal into electrical signal is illustrated in Supplementary Fig. S11.

### Self-powered vertical tribo-transistor with multi-sensing-memory function

**Fig. 4:**
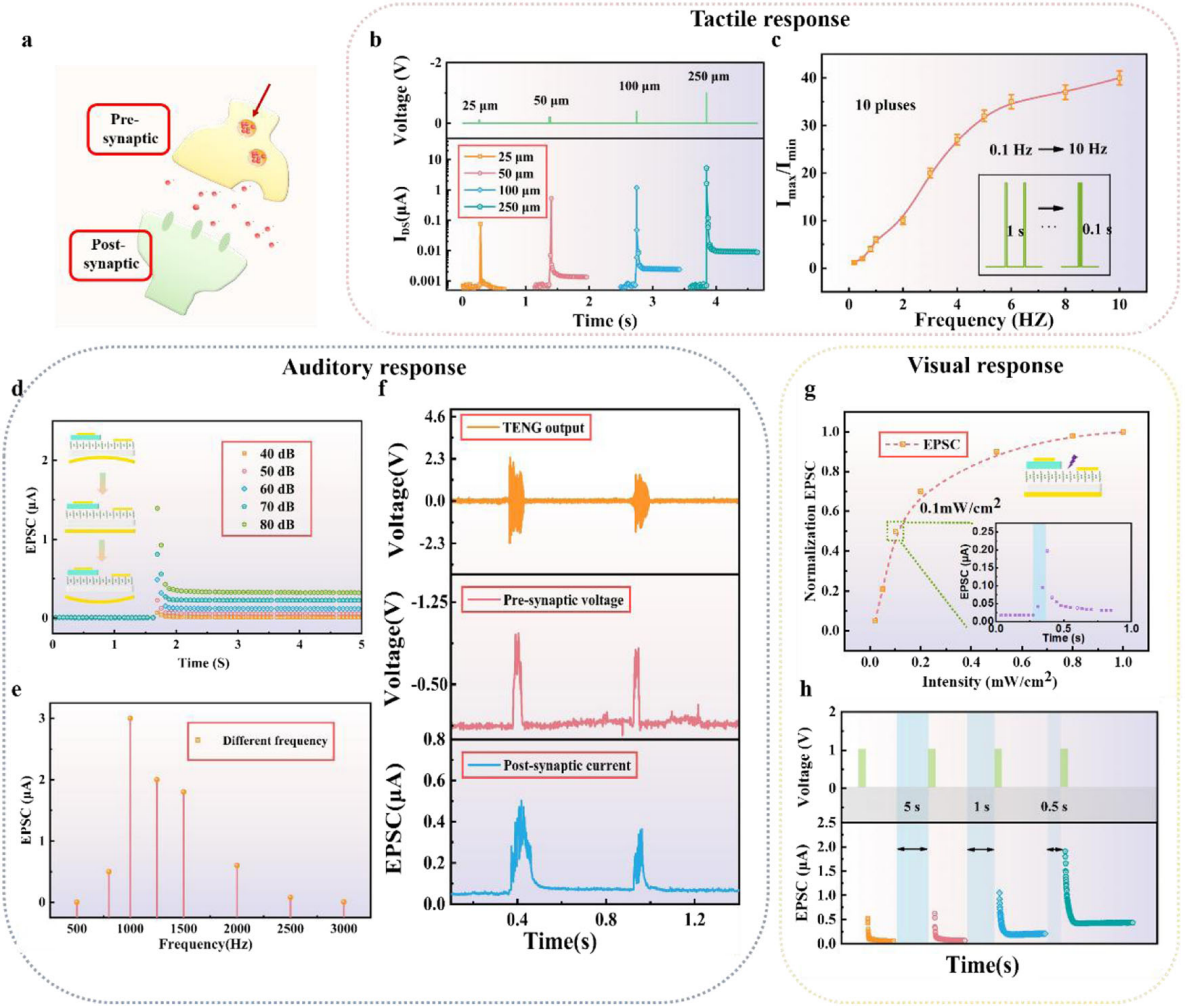
Synapse response of tactile, auditory, and visual. **a** The sensory transduction and memory-computing process. The tactile response of our VTT: **b** The EPSC with different distance and **c** I_max_/I_min_ with different pulse frequencies. The error bar in **c** means the value of I_max_/I_min_ within ten cycles. The auditory response of our VTT: **d** EPSCs of VTT with sound pressure level ranging from 40 dB to 80 dB. **e** EPSCs of VTT with different sound frequencies. **f** Recorded TENG output, pre-synaptic voltage and corresponding post-synaptic current. **g** The visual response of our VTT Normalization EPSCs with different light intensity. **h** EPSC amplitudes with two successive light pluses with different interval time.

Furthermore, the multi-sensing-memory function of our VTT is demonstrated. Note that EDL effect is generated at ion-gel layer, which is accompanied with retentive effects and the ion transport in EDL, resulting in synaptic behaviors of VTT. In our sensing-memory-computing system, the sensory transduction process can be regarded as the presynaptic process, and the memory-computing process can be regarded as the postsynaptic process, as depicts in Fig. 4a. We first study the synaptic plasticity of the VTT with different tactile, auditory, and visual input signals. Fig 4b, c show the tactile response of this VTT, where the gate voltage is controlled by the distance between gate electrode and IL, and the drain-source voltage is fixed at a constant value of −0.5 V. With the distance increased from 25 μm to 250 μm, the equivalent gate voltage is increased from 0.5 V to 1.5 V, and the post-synaptic current amplitude of I_DS_ is augmented from 0.1 μA to 8 μA, as shown in Fig. 4b. Meanwhile, the EPSC peak as a function of pulse time with a fixed distance of 25 μm is depicted in Supplementary Fig. 12. The EPSC firstly shows a linear enhancement when the pulse time is below 300 ms and then becomes saturated when the pulse time increases from 300 ms to 1000 ms. Supplementary Fig. 13 shows the responding time and decay time of the tactile EPSC, and the responding time is increased from 5 ms to 40 ms with the distance increased from 25 μm to 250 μm. Clearly, larger distance will result in longer time of gate electrode and ion-gel insulating layer to contact with each other and then induce more ions and carries. Moreover, paired-pulse facilitation (PPF) is also presented in Supplementary Fig. 14. As the interval time of two continuous pluses is shorter than the ion transfer time, an obvious increase of the second EPSC amplitude can be observed. Thus, we further investigated the EPSC amplitude under different pulse frequencies. As illustrated in Fig. 4c, with the frequency changed from 1 Hz to 10 Hz, I_max_/I_min_ (I_max_ is the 10th EPSC amplitude, I_min_ is the 1st EPSC amplitude) increases from 1.2 to 40, which is critical for future neuromorphic computing such as dynamic high-pass filter.

Additionally, for the auditory response, the EPSCs of the VTT with a sound pressure level ranging from 40 dB to 80 dB are shown in Fig. 4d. When the auditory pulse (100 ms) is applied to VTT, the EPSC rapidly reaches a peak value and then gradually decays to resting current. Meanwhile, the magnitude of EPSC increases from 0.5  μA to 3.2 μA when the sound pressure level increases from 40 dB to 80 dB, and the responding time and decay time of EPSC is also increased with the sound pressure level increased (Supplementary Fig. 12). Moreover, the effect of different sound frequencies (100 ms) on the EPSCs of the VTT is depicted in Fig. 4e. The EPSC amplitudes first increases and then decreases with the frequencies ranging from 500 Hz to 3000 Hz, and the EPSC reaches its peak at the frequency of 1000 Hz. Fig 4f shows the V_oc_ of TENG, transferred pre-synaptic voltage, and corresponding post-synaptic current is driven by two different voice signals. Clearly, different voice signals can be distinguished with different post-synaptic currents.

Furthermore, the visual response of VTT is illustrated in Fig. 4hg. The temporal response of normalized EPSCs as function of light intensity is demonstrated in Fig. 4h, and the inset shows a typical EPSC response with the intensity of 0.1 mW/cm^2^. The amplitudes of EPSC increase almost linearly within a small light intensity range below 0.1 mW/cm^2^, and then a saturated EPSC would be recorded with further increase of the light intensity. Meanwhile, the responding time and decay time are also shown in Supplementary Fig. 12. The working mechanism of VTT under light is shown in Supplementary Fig. 15. Figure 4g illustrates the application of two successive light pulses with different interval time (5 s, 1 s, and 0.5 s), where the second EPSC peak increases with the decreases of the interval time. The increased EPSC value after the first light pulse and the second (consecutive) light pulse are denoted as EPSC_1_ and EPSC_2_, respectively. Supplementary Fig. 16 depicts the ratio of EPSC_2_/ EPSC_1_ as a function of interval time, which is an indicator of enhancement of synaptic connections. The increased ratio suggests that the ion transport-mitigating layer contribute notably to maintain synaptic connections. Furthermore, VTT shows high endurance and stability with several cycles and days, respectively, as shows in Supplementary Fig. 17. These multi-sensing synaptic functions of our VTT lay foundation for realizing artificial intelligence system with sensing-memory-computing ability.

### Self-powered vertical tribo-transistor with multi-sensing-memory-computing (MSMC) function

**Fig. 5:**
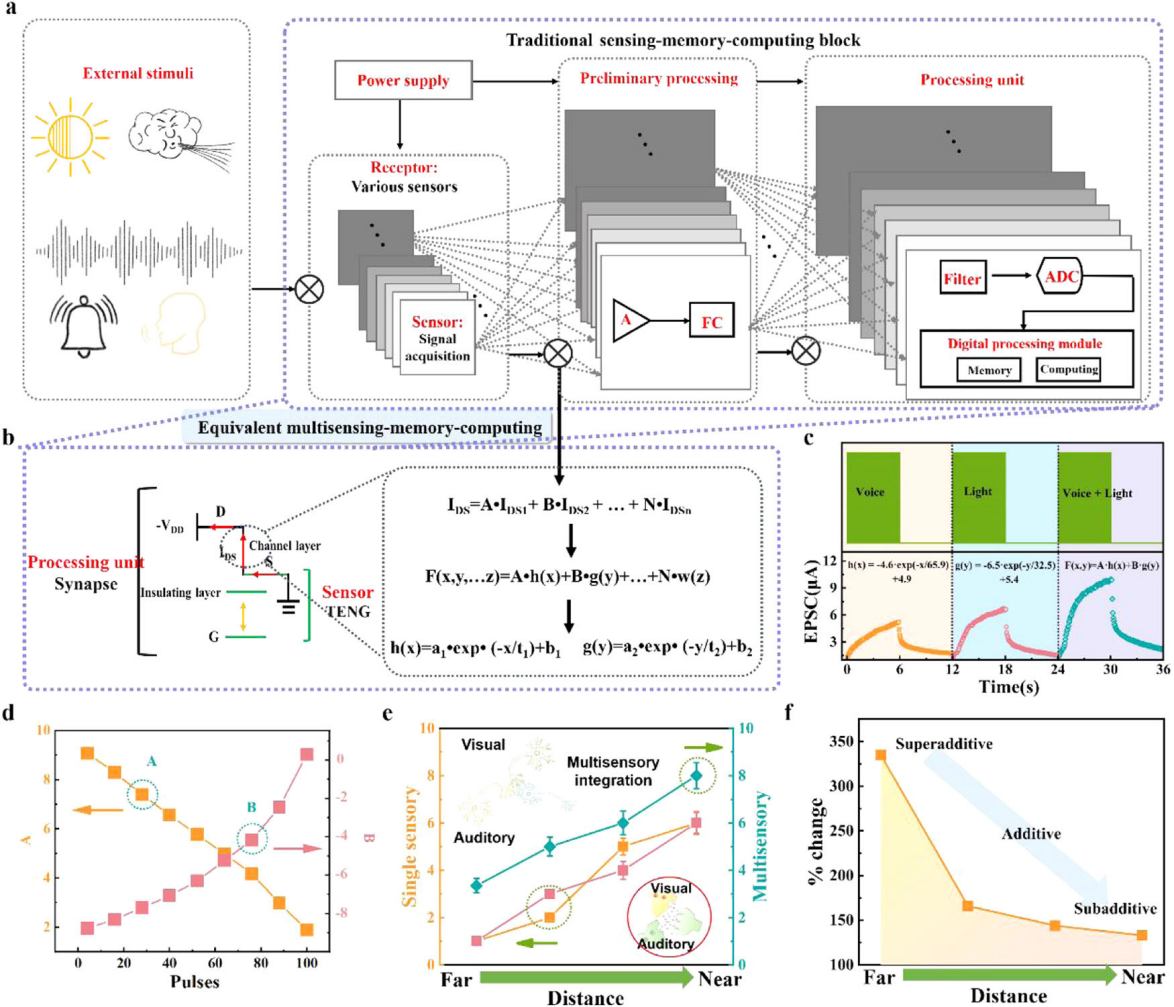
Multi-sensing-memory-computing model and mimicking superior colliculus. **a** External stimuli of our environment and traditional sensing-memory-computing block, which consists of power supply unit, receptor unit, preliminary processing unit, and processing unit. **b** Equivalent multi-sensing-memory-computing of our VTT and corresponding cross-correlation functions between single sensing-memory-computing and multi-sensing-memory-computing. **c** EPSC with different stimuli, which can be well-fitted by cross-correlation functions. **d** The change of A and B with increased external sensing pulses (single voice pulses, and light pulses; or voice+light pulses). **e** Single sensory stimuli and multisensory stimuli with different sensory strength. The error bar in (**e**) means the stimuli of single sensory and multisensory within ten cycles. **f** The %change from superadditive, additive, to subadditive.

Importantly, the function of MSMC in the human brain can associate and learn crossmodal information. Inspired by this fact, we then investigated the MSMC function of our VTT device. Figure 5a depicts the traditional digit MSMC system, which contains several components, such as the signal acquisition part (receptors), the power supply part, the preliminary processing parts, and the processing unit. However, the separated analog-digital hybrid circuit, memory, computing, convolution computation and other hardware result in slow processing speed and high power consumption. Our self-powered VTT device is illustrated in Fig. 5b, the TENG component in VTT is regarded as the sensor of the MSMC system, and VTT is regarded as the preliminary and processing unit. Since the working mechanism of VTT under mechanical and optical stimuli is different, such device is attractive for processing multi-sensing information without an extra preliminary processing unit. Here, the output current (I_DS_) of VTT is depicted as I_DS_ = A×I_DS1_ + B×I_DS2_ + … + N×I_DSn_, where I_DS1_, I_DS2_, and I_DSn_ are the EPSC with different external stimuli (visual, auditory, tactile, etc.). Meanwhile, the expression of I_DS_ is depicted as eq. (4):

4 $${{{{{\rm{F}}}}}}({{{{{\rm{x}}}}}},{{{{{\rm{y}}}}}},\ldots {{{{{\rm{z}}}}}})={{{{{\rm{A}}}}}}\times {{{{{\rm{h}}}}}}({{{{{\rm{x}}}}}})+{{{{{\rm{B}}}}}}\times {{{{{\rm{g}}}}}}({{{{{\rm{y}}}}}})+\ldots+{{{{{\rm{N}}}}}}\times {{{{{\rm{w}}}}}}({{{{{\rm{z}}}}}})$$

where h (x), g (x), and w (z) are I_DS1_, I_DS2_, and I_DSn_ of VTT, respectively, A, B, and N are constants. For example, as shown in Fig. 5c, the EPSC of VTT under auditory stimuli is fitted as h(x) = −6.5 × exp × (−x/65.9) + 4.9, and the EPSC of VTT under optical stimuli is fitted as g(y) = −6.5 × exp × (−y/32.5) + 5.4. Based on the change of EPSC amplitude, the response under auditory and optical stimuli simultaneously can be fitted as F (x,y) = A × h (x) + B × g (y). Noticeably, with the increases of stimuli pluses, the constant of A decreases, while B increases, as illustrated in Fig. 5d. We further investigate the MSMC function of VTT with tactile and visual response simultaneously, as illustrated in Supplementary Fig. 18. This phenomenon further demonstrates that our artificial multisensory device with the MSMC function could facilitate the parallel processing of large amounts of external stimuli information, which decreases the data exchange between storage and computation, increases working speed, and decreases power consumption compared with traditional CMOS architecture.

Furthermore, considering that multisensory integration is a critical and ongoing determinant of human behavior. Noticeably, inputs from different sensory neurons are converged on cells in the superior colliculus, and then result in multisensory integration. Here, we mimicked the response of superior colliculus in the human brain upon the approaching of a dog to demonstrate the multisensory integration (as illustrated in Supplementary Fig. 19). As the dog became closer, the individual information (visual and auditory) shows higher impulses, while VA responses became proportionately weaker (Fig. 5e). In addition, the change between single sensory and multi-sensory is defined as VA/(Vmax, Amax)×100% (%change). Clearly, the %change is enhanced with the dog being closer, as illustrated in Fig. 5f. The neural computation involved in their integration is changed from superadditive to additive and then to subadditive, and the detailed illustrations are provided in Supplementary Note 2. Moreover, as multisensory integration could enhance the single sensory signal and the behaviors that depend on them, we further constructed an artificial stimulus-response system to further prove the multisensory enhancement concept. The robot hand system supported by our VTT is also illustrated in Supplementary Note 3.

### Self-powered vertical tribo-transistor with MSMC function for multi-mode emotion recognition

**Fig. 6:**
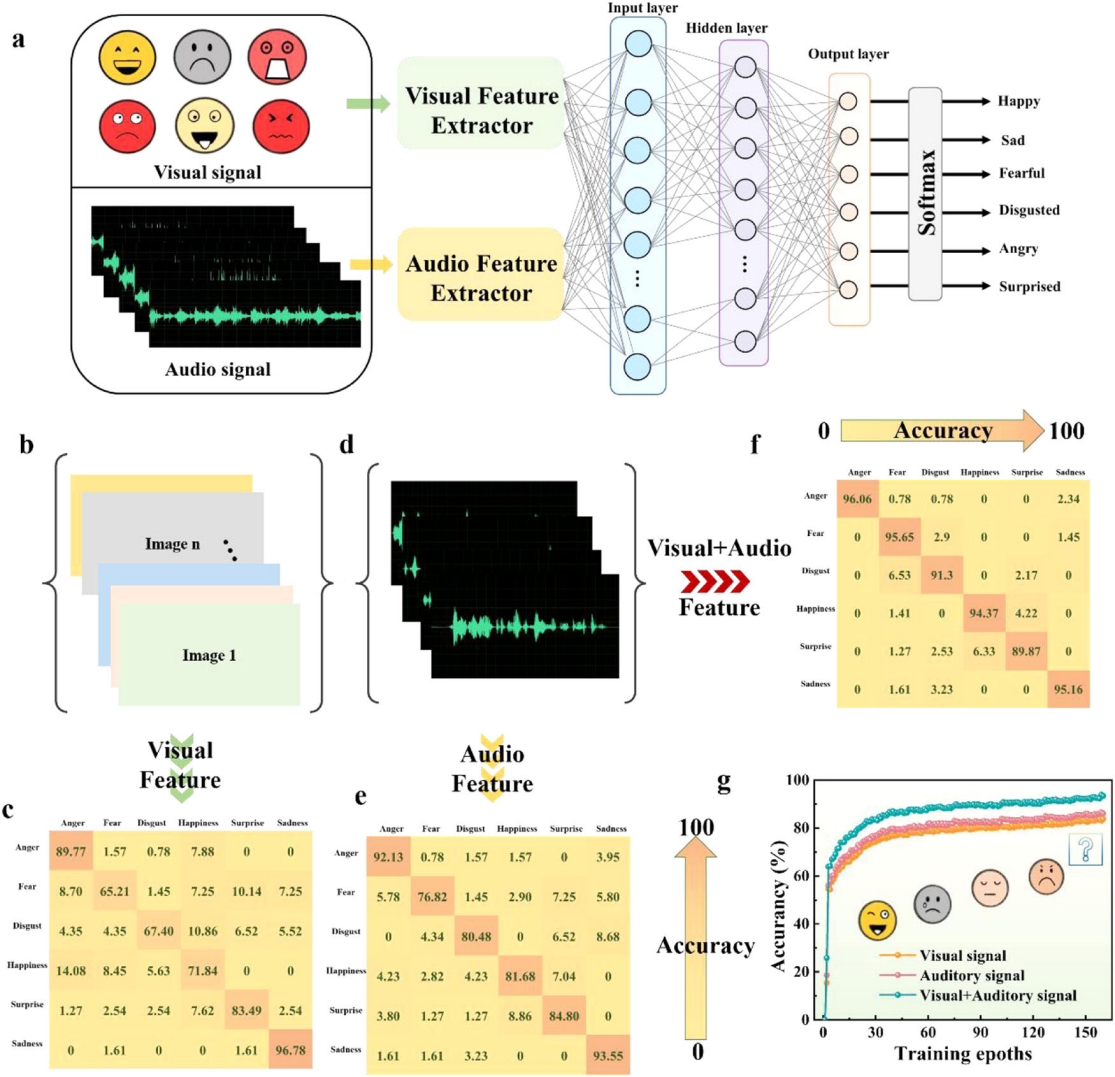
Multi-model emotion recognition system. **a** The schematic diagram of multi-model emotion recognition system to recognize the six emotions (anger, fear, disgust, happiness, surprise, and sadness), where the recognition process includes information fusion, feature extract, data classification, emotion recognition. **b** The pictures extract from database without sound information for emotion recognition. **c** The recognition accuracy of different emotion with single visual model. **d** The sound extract from database without visual information for emotion recognition. **e** The recognition accuracy of different emotion with single auditory model. **f** The recognition accuracy of different emotion with multi-model model. **g** The recognition accuracy of our multi-model emotion recognition system at different model.

All of the above characterizations and analyses imply that our VTT exhibits excellent MSMC function in a single device. Thus, based on the MSMC function, we construct a multi-model emotion recognition system to further demonstrate the capability of VTT in extending artificial intelligent boundary. Noticeably, the emotion is tightly entangled with behavior of human, while the complete emotion information cannot be obtained only through visual or auditory perception. Thus, combining the characteristic information and extracted feature information from the two perceptions would improve the emotion recognition accuracy and reliability. As depicts in Fig. 6, through single VTT device, we achieve the multi-model emotion recognition. Noticeably, the effective features are extracted from visual and auditory mode by our devices (the EPSC with different light intensity is been separated into 16 states, and each state corresponds to 16 numerical values of colorful images, as depicted in Supplementary Fig. 22), and then the information is transferred to input layer for further processing, as indicates in Fig. 6a and Supplementary Fig. 23. Here, the select fusion model is data-level fusion, which directly combines the most original data collect by our devices without special processing to construct a group of new data. Then the several basic discrete emotions, such as sadness, fear, disgust, surprise, excitement and anger can be recognized by operation methods to compute and process data from multiple data sources.

We first demonstrate the motion recognition accuracy with visual model, auditory model, and multi-model, respectively, as illustrates in Figs. 6b–f. Several images are selected from a video without sound information, sound wave, or visual information, and the recognition accuracy of six emotions is summarized at Figs. 6c, e, and 6f. Note that, the accuracy of multi-model emotion recognition is significantly higher than that of a single model, which further indicates that our multi-model emotion recognition system is suitable for recognizing the complex emotional category through a certain combination mode. Thus, the comparison of recognition accuracy between different models is further shown in Fig. 6g. Clearly, the visual + auditory model exhibits the highest recognition accuracy of 94.05% after 140 epochs, while the accuracy of other single models is lower than that of the multi-model system. Moreover, the accuracy is higher than the values reported in previous works as illustrates in Table 1. These results apparently demonstrate that MSMC VTT device can well retain the data information on each modal sensor to avoid the loss of information, maintain the integrity of information, and significantly enhance the certainty of emotion.

**Table 1:** Accuracy of multi-modal recognition with different model

Multi-model	Model	Mode	Device number	Accuracy	Ref.
Visual+Auditory	Emotion	Software	/	90.8%	^48^
Visual +Auditory	Emotion	Software	/	77.5%	^49^
Visual + Auditory	Emotion	Software	/	85.9%	^50^
Visual+Auditory	Emotion	Software	/	82.9%	^51^
Visual+Auditory	Emotion	Software	/	80.3%	^52^
Visual+Tactile	Digit	Device	Two	90%	^53^
Visual+Tactile	Digit	Device	Two	86.8%	^19^
Visual+Tactile	Alphabet Letters	Device	/	92%	^24^
Visual+Auditory	*Emotion*	*Device*	*Single*	*94.05%*	*This work*

Finally, the superiority of our self-powered multi-sensing-memory-computing device is concluded. (i) simple structure and self-powered device: the receptors and synapses are integrated in a single device based on a simple configuration of VOFET without any redundant layers, which enhances packing density, simplifies fabrication procedures and reduces chip cost, and further decreases power consumption with the self-power function of TENG. (ii) high sensitivity: The amplification, low SS (91.8 mV/dec) and high current density (5 mA/cm^2^) of VTT ensure high sensing sensitivity (the tactile sensitivity improves 711 times over individual TENG). (iii) high efficiency and conversion speed: the multi-sensing-memory-computing function and the interaction of multifunction integration of our VTT increases the conversion speed, which is associated with the decreased physical separation between sensory receptors and processed nodes compared with the traditional CMOS architecture. (iv) high recognition accuracy: the multi-model emotion recognition is successfully achieved based on the multi-sensing-memory-computing ability of our VTT, which significantly improves the computing functionality, recognition variety and accuracy.

## Discussion

In summary, we experimentally demonstrate a multi-sensing-memory-computing device that integrated a TENG and a transistor in a single device with the configuration of VOFET. Compared with a single TENG device, the sensing sensitivity is significantly enhanced due to the excellent SS and high current density of VTT. Meanwhile, in addition to tactile and auditory perception, the visual perception can be mimicked with the unique advantage and optical-sensitivity performance of MXene electrode. By calculating the cross-correlation functions, the memory-computing between multi-sensing is demonstrated, and the superior colliculus function and artificial stimulus-response system is also mimicked thanks to the multi-sensing integration property of our VTT. Finally, a multi-model emotion recognition system based on our VTT is achieved, which enables 94.05% recognition accuracy of emotion via data-level model fusion. This proof-of-concept work realized tactile/auditory/visual sensing-memory-computing within a single device, which has the potential to decrease the data movement between sensor, memory, and computing units, and paves the way for brain-inspired computing paradigms.

## Methods

### Device fabrication

The prepared ion–gel solution was spin-coated on Si substrate as the friction layer and insulation layer of TENG and VOFET, respectively. The ion–gel solution was obtained by mixed PAN, [Li^+^TFSI^−^], EC, and PC in a weight ratio of 7:1:1:1 and then the mixture was magnetically stirred at 500 rpm for 6 h. Then, the 2 mg/ml MXenes aqueous solution was selected as the top electrode and source network electrode of TENG and VOFET, respectively. Next, the prepared PDVT-10 solution was spin-coated on the electrode layer as the semiconductor layer, and then the patterned source and drain electrodes were thermally evaporated through a shadow mask. Finally, the fabricated device was transferred to the thermally evaporated gate electrode as VTT device.

### Device characterization

The output performance of TENG was measured by an oscilloscope. The electrical performance of the synaptic transistor was characterized by Keithley 4200. The surface morphology of MXenes was examined by using scanning electron microscopy (SEM, Verios G4), transmission electron microscopy (TEM, Tecnai G2 F20). The UV-Vis absorption spectra of the films were measured by ultraviolet–visible near infrared spectrophotometer (Shimadzu UV-3600 Plus).

## Supplementary information

Supplementary Information

## Data Availability

The data that support the findings of this study are available from the corresponding author upon request.

## Code availability

The codes used for the simulations are available in [https://github.com/YaqianLiuFZU/NCOMMS-22-33550]^47^.
