# Supplementary material for: Self-powered high-sensitivity all-in-one vertical tribo-transistor device for multi-sensing-memory-computing
Source: Nat Commun. 2022 Dec 23;13:7917. doi: 10.1038/s41467-022-35628-0 (PMC9789038; doi:10.1038/s41467-022-35628-0)
Supplement: Supplementary file 1 — Supplementary Information [file 41467_2022_35628_MOESM1_ESM.pdf]

Supplementary information

Self-powered High-sensitivity All-in-one Vertical tribo-transistor Device for multi-sensing-memory-computing

Yaqian Liu[1,2], Di Liu[1,3], Changsong Gao[1,3], Xianghong Zhang[1,3], Rengjian Yu[1,3], Xiumei Wang[1,3], Enlong Li[1,3], Yuanyuan Hu[4], Tailiang Guo[1,3], Huipeng Chen[1,3*]

[1]Institute of Optoelectronic Display, National & Local United Engineering Lab of Flat Panel Display Technology, Fuzhou University, Fuzhou 350002, China

[2]School of Physics and Electronic Engineering, Zhengzhou University of Light Industry, Henan 450002, China

[3]Fujian Science & Technology Innovation Laboratory for Optoelectronic Information of China, Fuzhou 350100, China

[4]State Key Laboratory for Chemo/Biosensing and Chemometrics, School of Physics and Electronics, Hunan University, Changsha 410082, China

Email: hpchen@fzu.edu.cn

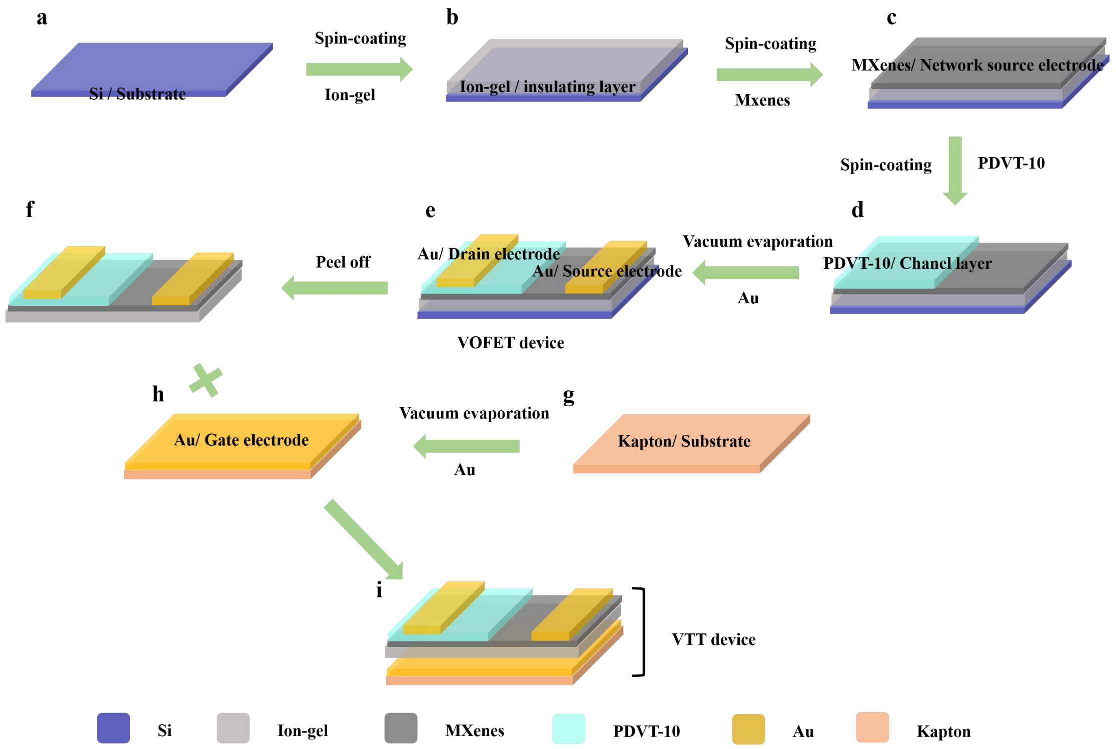

Supplementary Figure 1 The detailed fabrication process of VTT. (a) and (b) Ion-gel solution was spin-coating on Si substrate as insulating layer of VTT; (c) MXenes solution was spin-coating on insulating layer as network source electrode; (d) PDVT-10 solution was spin-coating on network source electrode layer as channel layer, and then the device was partially immersed in coloroform solution by a dip way to pattern PDVT-10; (e) Au electrode was thermally evaporated through a shadow mask as source and drain electrode; (f) Peeled of device from Si substrate as the top part of VTT; (g) and (h) Au electrode was thermally evaporated on the Kapton substrate as the gate electrode of VTT; (i) Integrated (f) and (h) as VTT device.

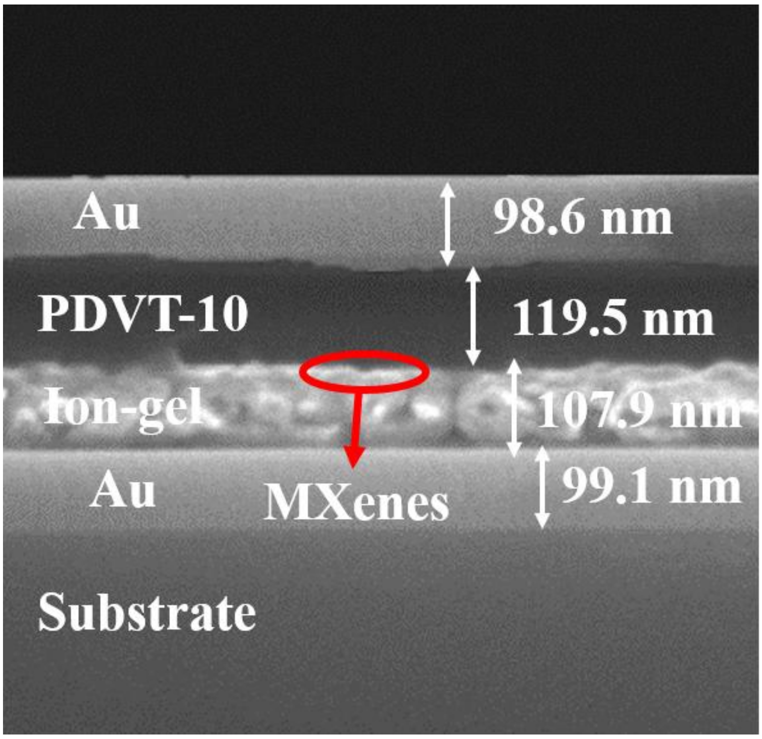

Supplementary Figure 2 The cross-section SEM of vertical tribo-transistor (VTT).

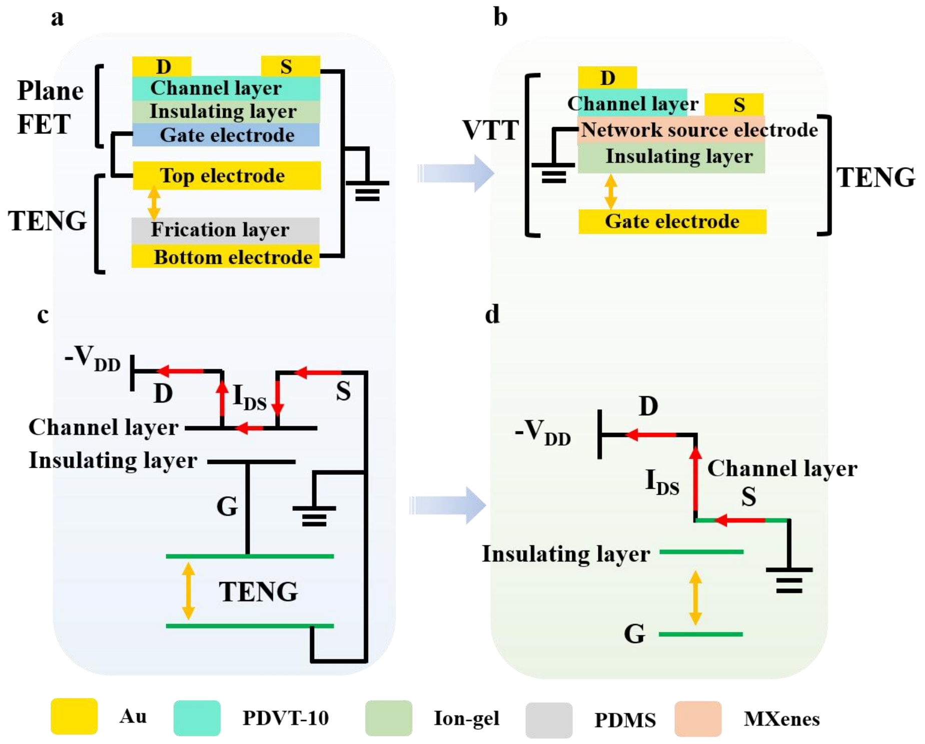

Supplementary Figure 3 The equivalent circuit of traditional TENG and planer transistor.

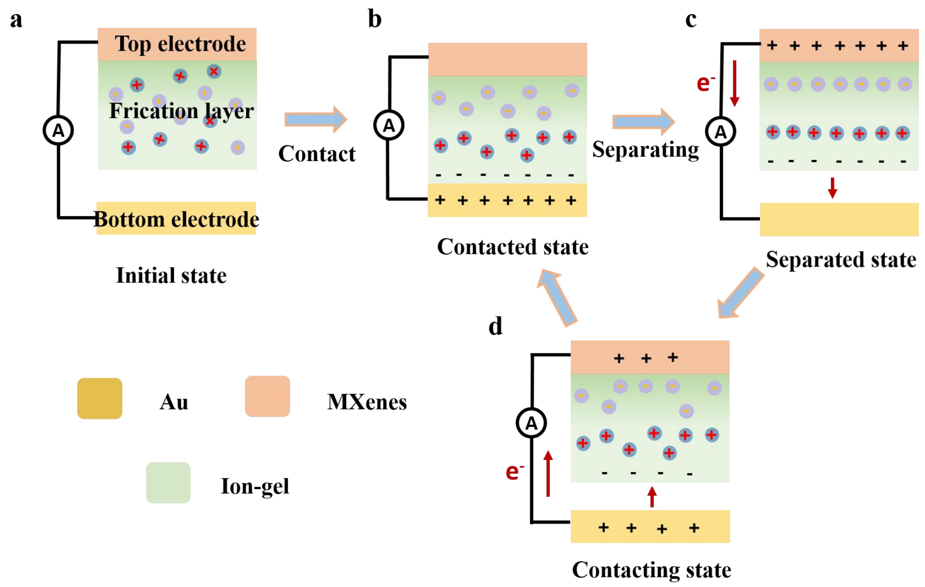

Supplementary Figure 4 The detailed working mechanism of TENG.

Supplementary Note 1

The triboelectric property of TENG was determined by the tribocharge surface density of the frication layer, which can be expressed by eqn. (1)[1]:

$$V_{OC} = \frac{\sigma d}{\varepsilon} \qquad (1)$$

According to equ (1), it was clearly shown that V_{OC} is dependent on ε, and an increase of the ε value results in an increase of V_{OC}. Meanwhile, the ε was dependent on C, which can be expressed by equ. (2):

$$\mathrm{C} = \frac{\varepsilon S}{4\pi k d} \qquad (2)$$

According to equ (2), it was clearly that higher capacitance of EDL can remarkably improve the performance of TENG.

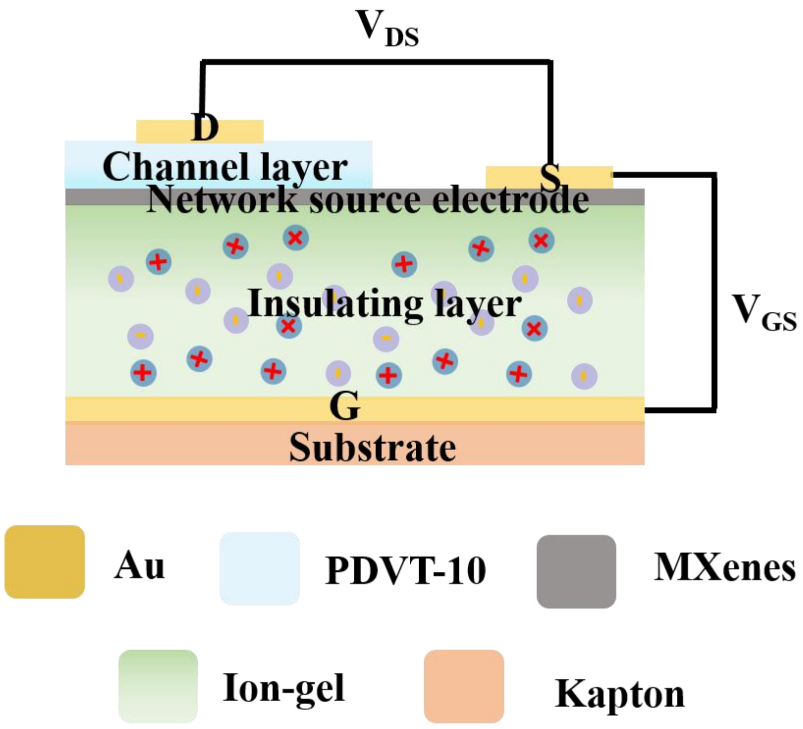

Supplementary Figure 5 The working state of VTT with external gate voltage.

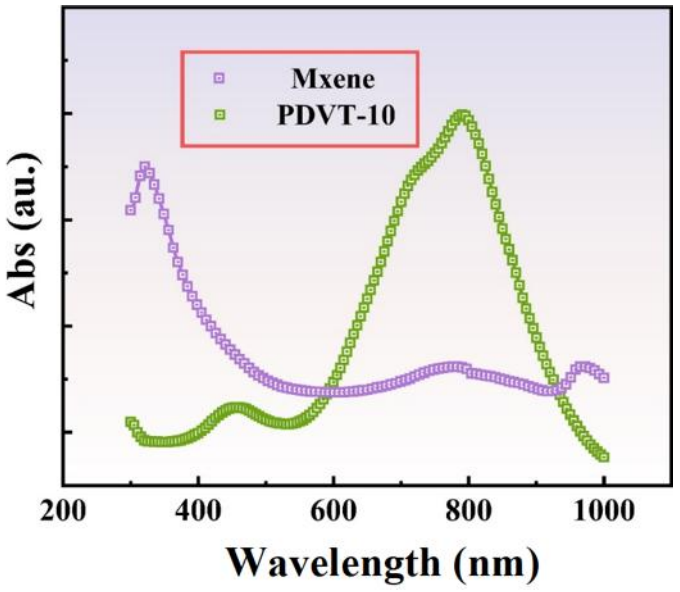

Supplementary Figure 6 The absorbance of PDVT-10 layer, MXenes, and MXenes/Kapton.

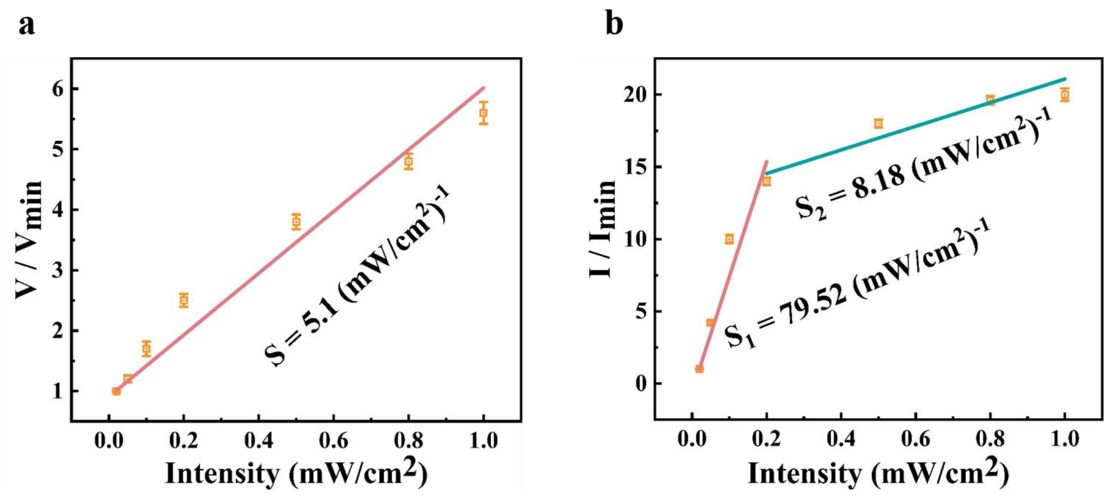

Supplementary Figure 7 The visual sensitivity of individual TENG and VTT. The error bars mean the values of V/Vmin or I/Imin within 10 cycles.

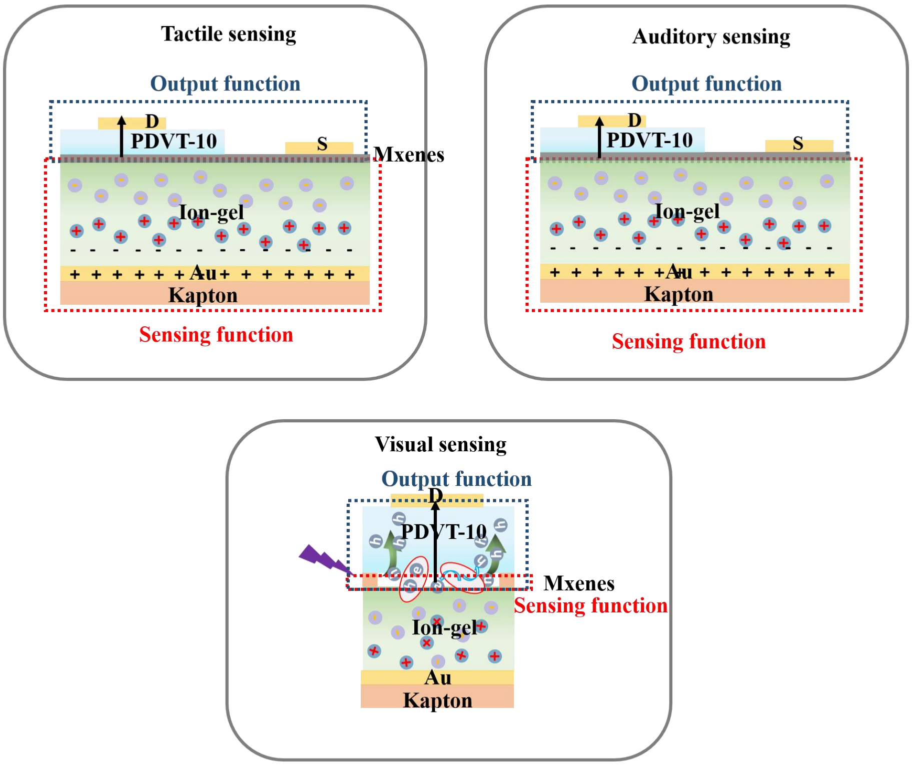

Supplementary Figure 8 Different sensing materials/areas on the VTT device play different roles in different perception.

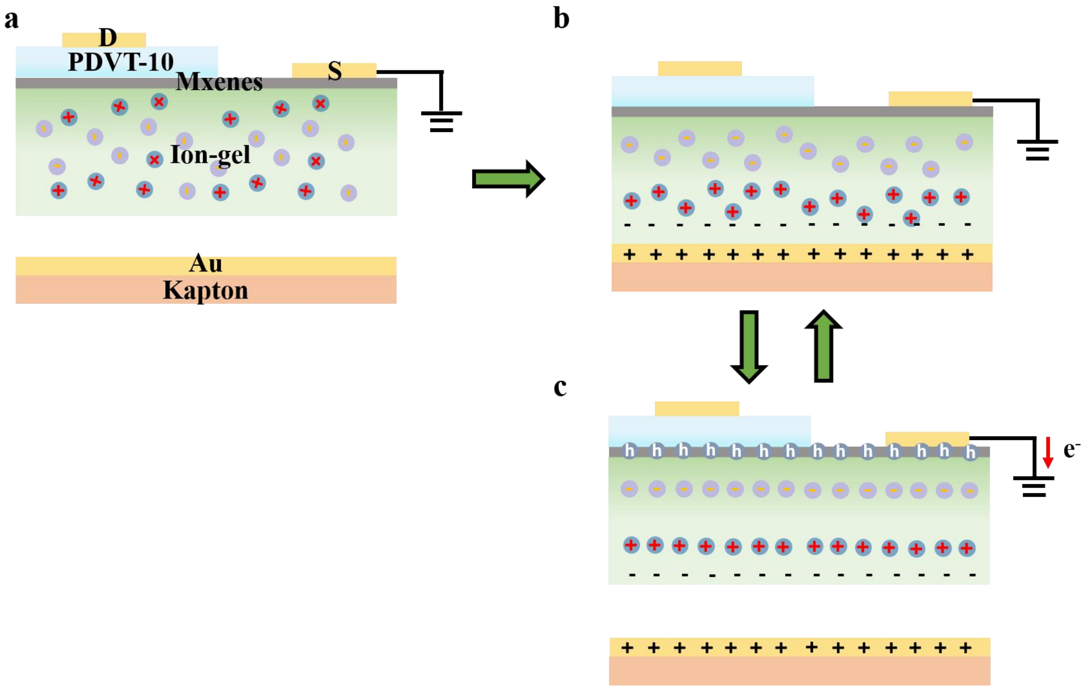

Supplementary Figure 9 The schematic illustration of initial state of VTT with gate electrode separated.

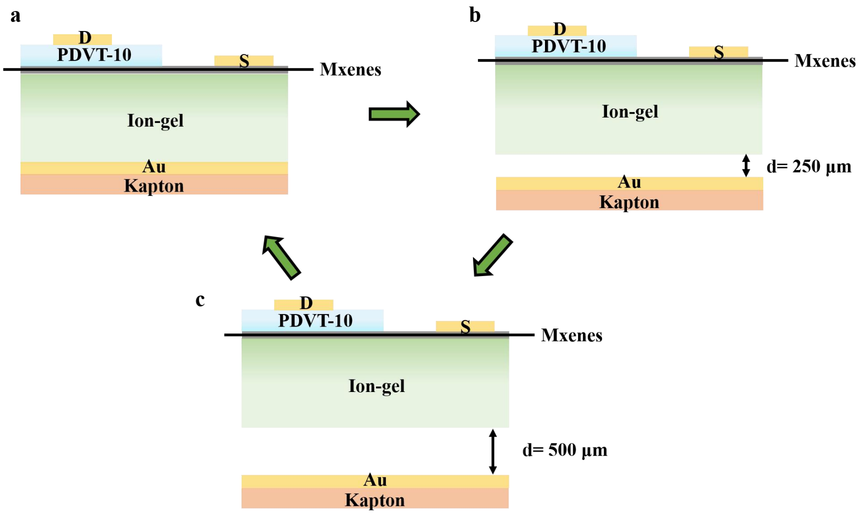

Supplementary Figure 10 The position of 1D potential distribution.

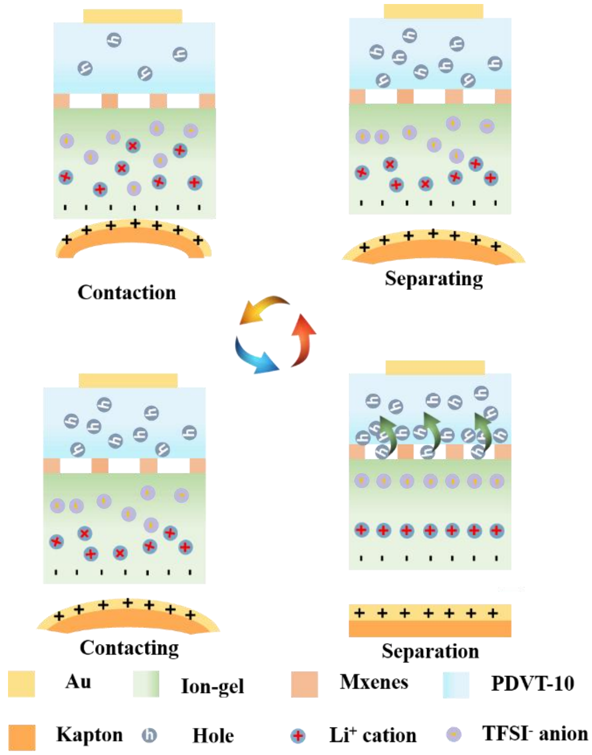

Supplementary Figure 11 Transform process of acoustic signals into electrical signals during the auditory sensing.

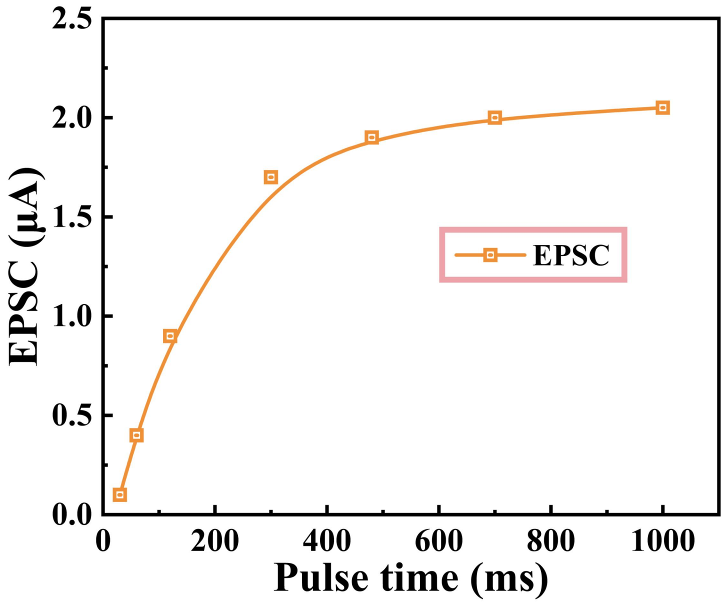

Supplementary Figure 12 EPSC peak as a function of pulse time with a fixed distance (25 μm).

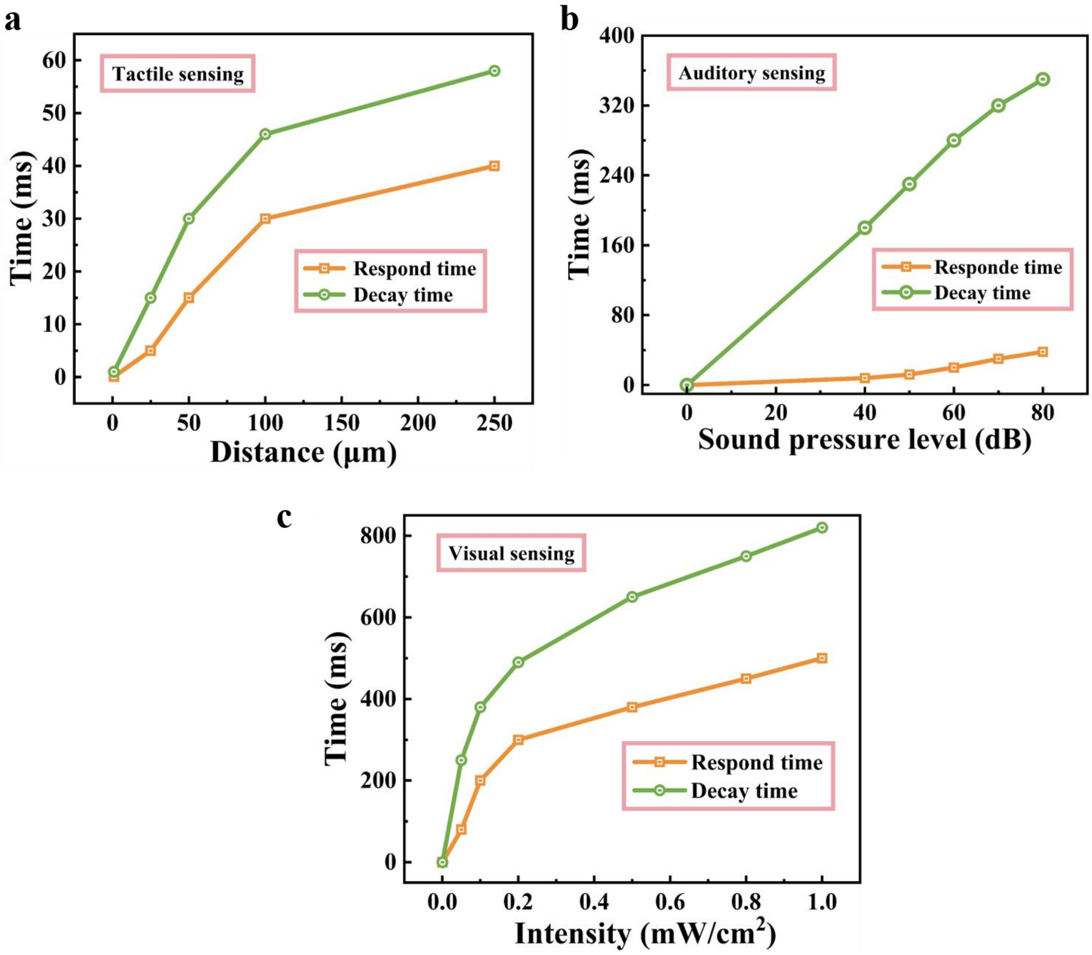

Supplementary Figure 13 The responding time and decay time of different sensing EPSC.

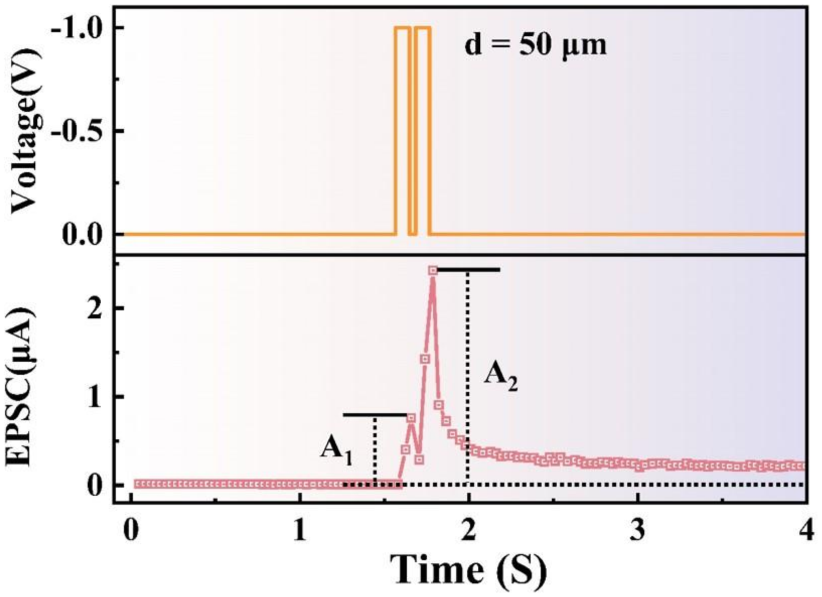

Supplementary Figure 14 Paired-pulse facilitation (PPF) of VTT with two successions tactile response.

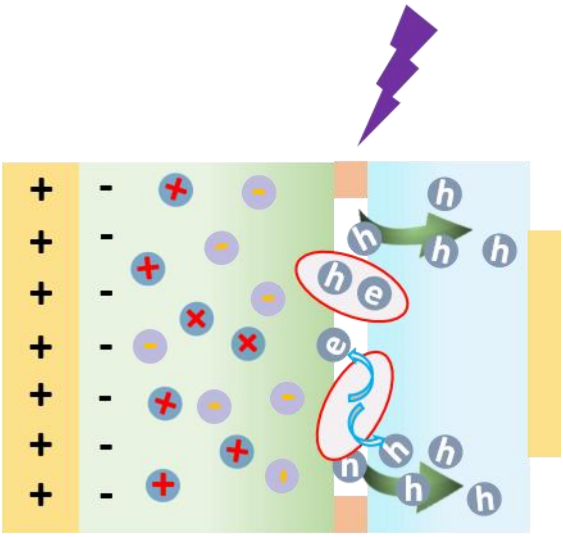

Supplementary Figure 15 The working mechanism of VTT synaptic performance with light. The network source electrode of VTT always with a TiO_2 oxidation layer formed on the Mxenes surface. Thus, TiO_2 would generate photo-excitons and exhibit a light response once exposed to 350 nm light. Then, the photo-induced holes generated within in TiO_2 would transfer to the PDVT-10 layer, which resulted in EPSC of VTT.

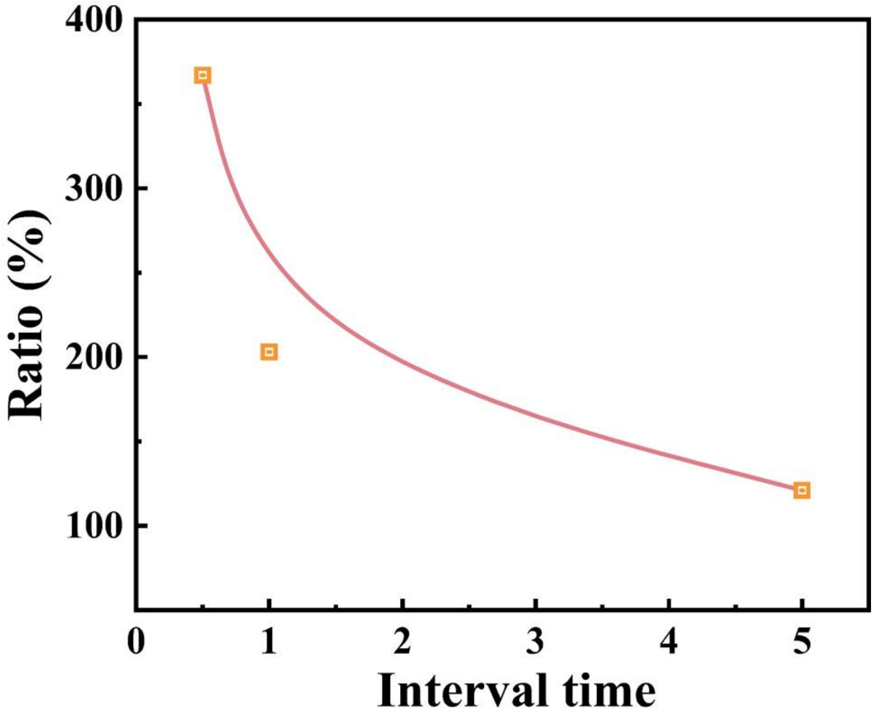

Supplementary Figure 16 The ratio of $EPSC_1$ to $EPSC_2$ as a function of light pluses interval time.

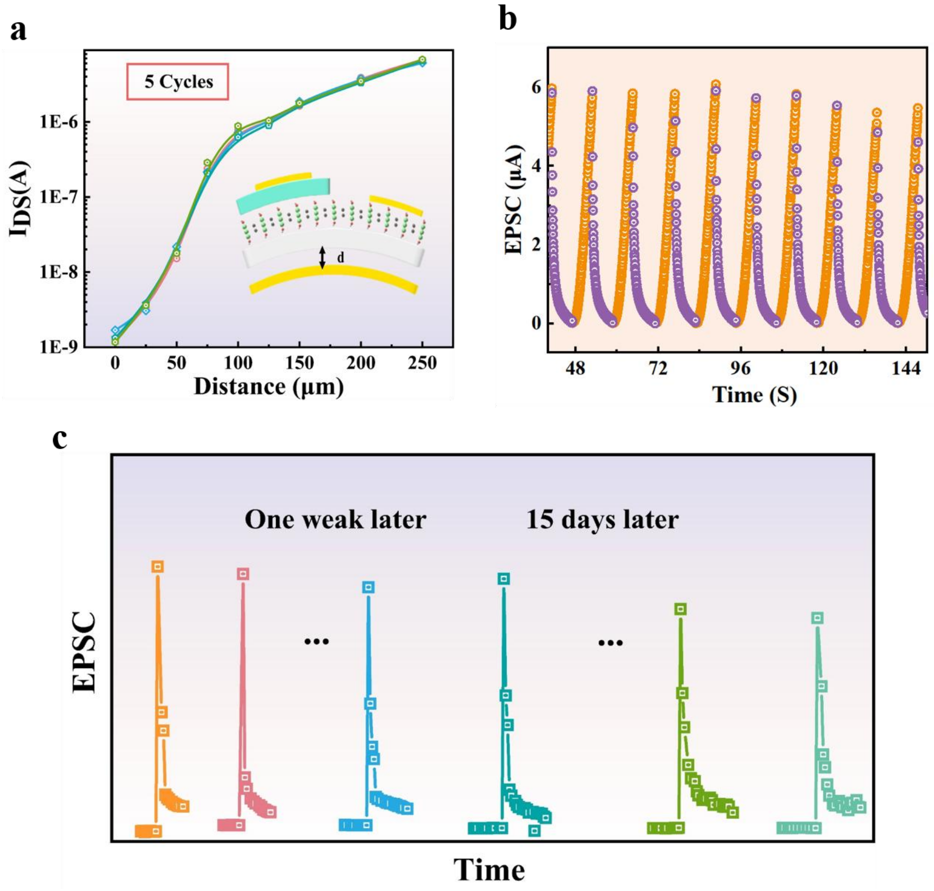

Supplementary Figure 17 The endurance and stability of VTT.

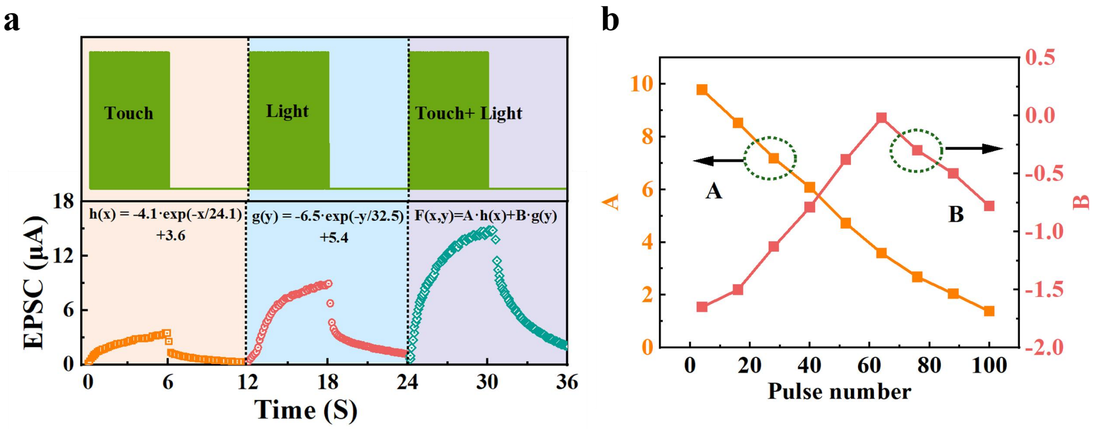

Supplementary Figure 18 The multi-sensing-computing function of VTT with light stimulation and tactile movement simultaneously.

Supplementary Note 2. The schematic diagram of multisensory integration.

The most fundamental manifestation of integration is multi-sensory enhancement, and the greatest enhancement always occurs for multisensory combinations of the weakest sensory stimuli. Hence, we further mimicked the superior colliculus in the human brain to demonstrate the multisensory integration. As illustrated in Fig. S19, the approaching of a dog was detected, based on sight and sound. When the visual (V) and auditory (A) signals were weakened (when the dog is far away), the impulses of V and A was regarded as one impulse，while 3.35 impulse of multisensory integration (VA) could be recorded (**Supplementary Figure 19**).

When the visual and auditory signals were weakened (when the dog is far away, **Supplementary Figure 19a**), the impulses of visual (V) and auditory (A) was regarded as one impulse, respectively while 3.35 impulse of multisensory integration (VA) could be recorded (**Supplementary Figure 19d**). **Supplementary Figure 19d** also illustrated the change between signal sensory and multisensory, which can be defined as VA/(Vmax, Amax) × 100%. 335% change can be calculated from the recorded impulses, which indicated that the neural computation involved in their integration is superadditive. Such response not only exceeded the most vigorous component response, but also exceeded their sum. As the dog became closer (**Supplementary Figure 19b** and **Supplementary Figure 19e**), the individual information (visual and auditory) shows higher impulses, while VA responses became proportionately smaller (**Figure 5e**). In addition, the impulses and their change were shown in **Supplementary Figure 19c** and **Supplementary Figure 19f**, where the

computation now became additive and then subadditive. Meanwhile, the % changes were 166% and 120% of additive and subadditive states, as illustrated in **Figure 5f**. Although both the additive and the subadditive computations also produce responses that exceed the most vigorous component response (that is, they all exhibit multisensory integration), their enhancements are proportionately less than the one of superadditive. All enhancements increase the probability of orientation, while the benefits of multisensory integration are proportionately greatest when cross-modal cues are weakest.

All enhancements increase the probability of orientation, while the benefits of multisensory integration are proportionately greatest when cross-modal cues are weakest.

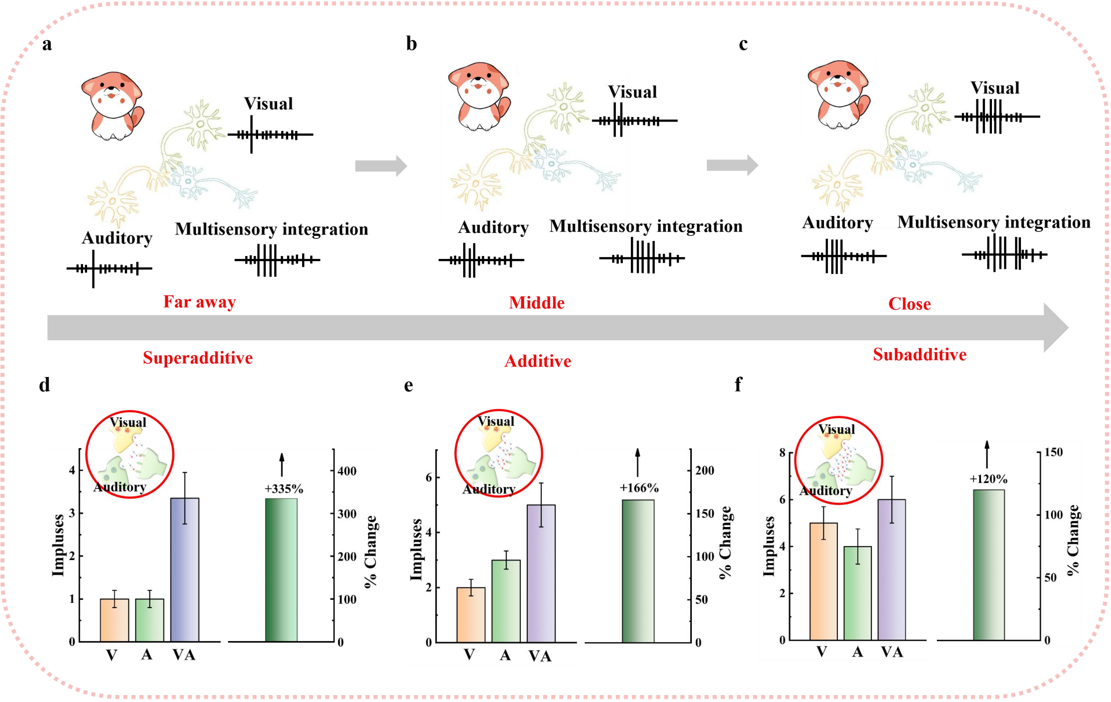

Supplementary Figure 19 The VTT deals different sensing information. The error bars in d-f means the values impulses and %changes within 10 cycles.

Supplementary Note 3. The schematic diagram of artificial stimulus-response system

Notably, as multisensory integration could enhance the single sensory signal and the behaviors that depend on them, we further constructed an artificial stimulus-response system to further prove the multisensory enhancement concept. The artificial stimulus-response system contained a VTT, an I-V conversion module, and a robot hand, and its corresponding circuit diagram are illustrated in **Supplementary Figure 20**. Different sensory stimulus response processes (visual and auditory) began begin with the post-synaptic current of VTT (I_{post}), then converted convertthis stimulus into an electrical pulse by the I-V conversion module (V_{out}), and finally the transmitted signal is transported to the robot hand. The robot hand deflection angle and active angle of robot hand are regulated by the pulses number of light or sound signals, as depicted in **Supplementary Figure 21**.

As shown in **Supplementary Figure 21**, with 30 succession light pulses actuated VTT, the robot hand could be activated at the 25th light pluses, and the deflection angle was changed from 120° to 110°. And the time of activation of the robot hand was 5.6s, which continued from 5.6 s to 6.1 s. Meanwhile, with 30 succession sound pulses actuated on VTT, the robot hand could be activated at the 20th light pluses, and the deflection angle can be changed from 120° to 105°. The time of activation of the robot hand was 5.2 s, which continued from 5.2 s to 6.2 s. However, with 30 succession sound and light pulses actuated on VTT simultaneously, the robot hand could be activated at the 12th light pluses, and the deflection angle was changed from

120° to 92°. The time of activation of the robot hand was 3 s, which continued from 5.6 s to 7 s. This result demonstrated that our artificial neural system is able to emulate a conscious response and significantly improves the response time after learning from repeated light stimuli. Note that the pulse-based operation of our system enabled lower energy consumption compared to all-CMOS–based circuits. This system is able to emulate a conscious response and significantly improves the response time after learning from repeated light stimuli.

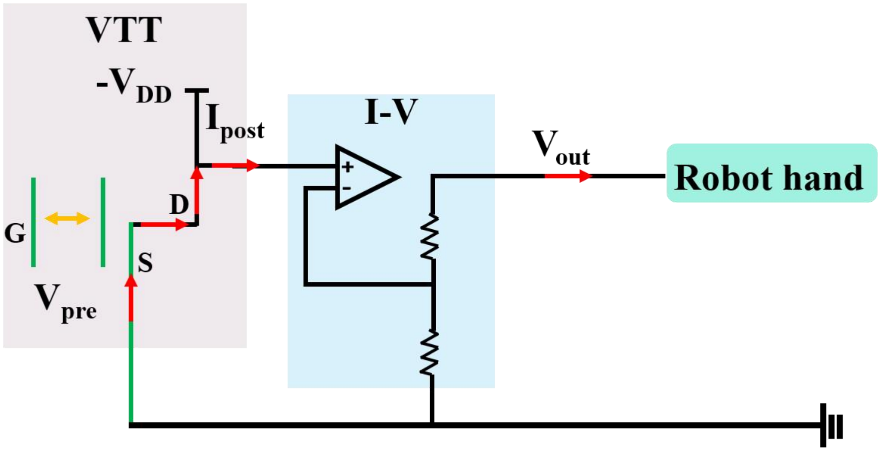

Supplementary Figure 20 The circuit diagram of artificial stimulus-response system.

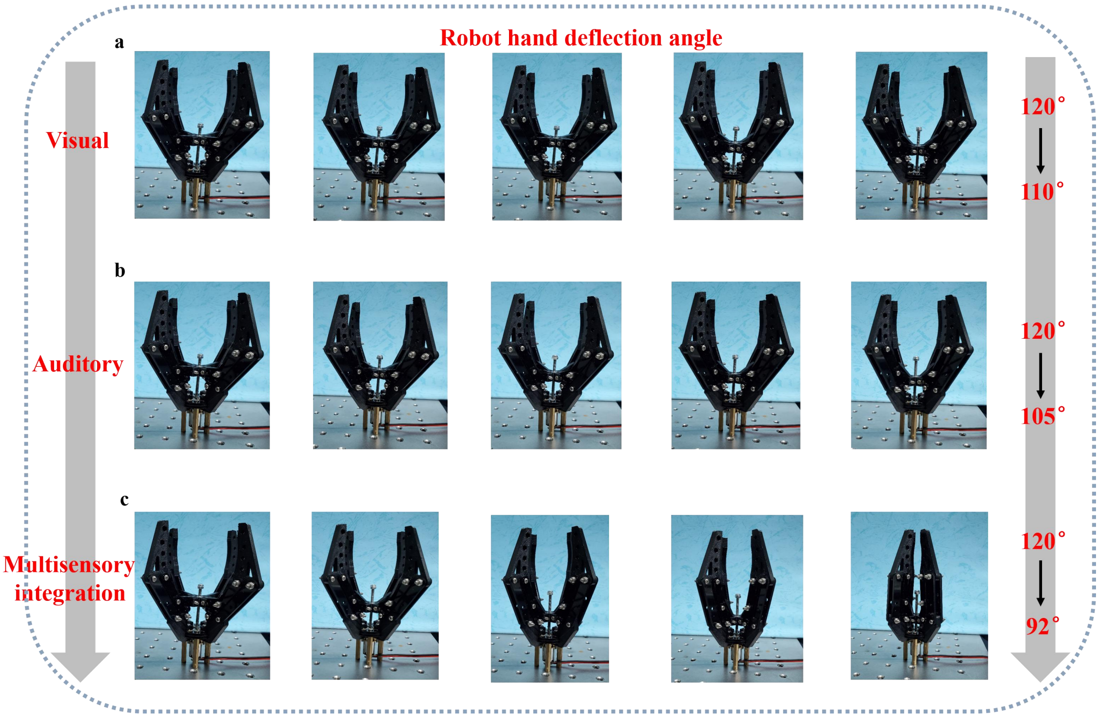

Supplementary Figure 21 Different sensory stimulus response process of our artificial stimulus-response system.

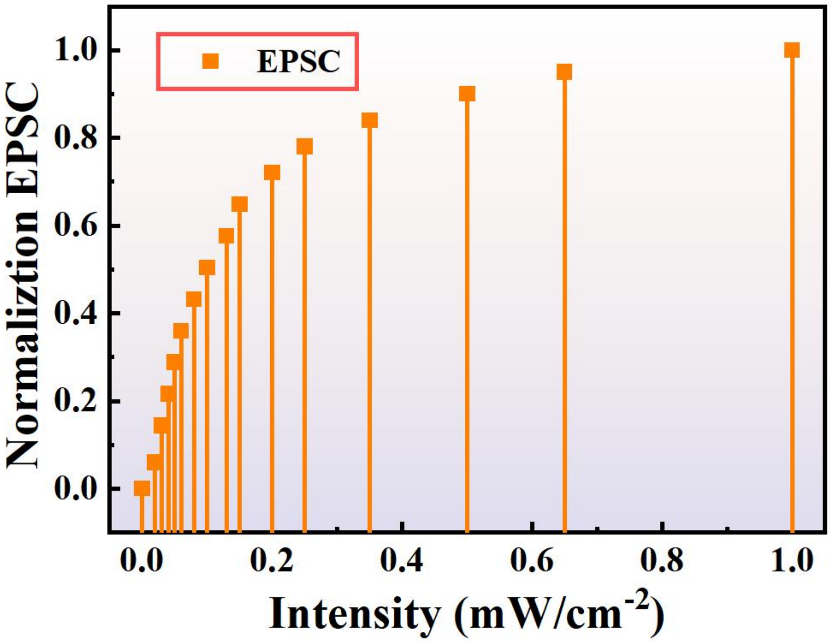

Supplementary Figure 22 Normalization EPSC peak of 16 different states.

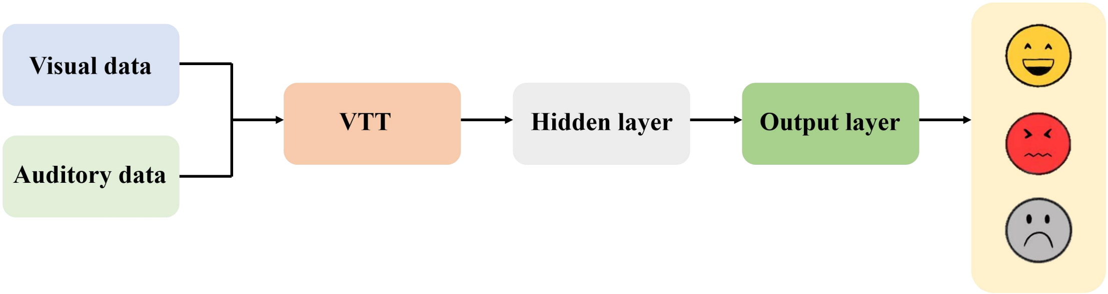

Supplementary Figure 23 Multi-model emotion recognition process.

References:

1. Niu, S. et. al, Theoretical study of contact-mode triboelectric nanogenerators as an effective power source. *Energy Environ. Sci.* 6, 3576–3583 (2013).
